# Assessing the impact of energy coaching with smart technology interventions to alleviate energy poverty

**DOI:** 10.1038/s41598-024-80773-9

**Published:** 2025-01-13

**Authors:** Joseph Llewellyn, Titus Venverloo, Fabio Duarte, Carlo Ratti, Cecilia Katzeff, Fredrik Johansson, Daniel Pargman

**Affiliations:** 1 Sustainable Development, Environmental Science and Engineering Department, KTH Royal Institute of Technology, Teknikringen 10b, Stockholm, 114 28 Sweden; 2 Senseable City Lab, Massachusetts Institute of Technology, 9-216 77 Massachusetts Avenue, Cambridge, MA 02139 USA; 3 Amsterdam Institute for Advanced Metropolitan Solutions, Gebouw 027W, Kattenburgerstraat 5, Amsterdam, 1018 JA Netherlands; 4 Media Technology and Interaction Design, School of Electrical Engineering and Computer Science, KTH Royal Institute of Technology, Lindstedtsv. 3-5, Stockholm, 100 44 Sweden

**Keywords:** Energy poverty, intervention, income, consumption, efficiency, smart-technology, Psychology and behaviour, Energy and behaviour, Energy efficiency, Energy justice, Energy policy, Energy security

## Abstract

Energy poverty affects 550,000 homes in the Netherlands yet policy interventions to alleviate this issue are rare. Therefore, we test two energy coaching interventions in Amsterdam: a static information group (*n* = 67) which received energy efficient products and one energy-use report, and a smart information group (*n* = 50), which also had a display providing real-time feedback on energy-use. Results across both groups, show a 75% success rate for alleviating energy poverty. On average homes reduced monthly electricity consumption by 62 kWh (33%), gas by 41 m^3^ (42%), bills by €104 (53%) and percentage of income spent on energy from 10.1% to 5.3%.

**Supplementary Information:**

The online version contains supplementary material available at 10.1038/s41598-024-80773-9.

## Introduction

### Energy poverty in the EU

Energy poverty affects 50 million homes within the European Union^1^. The Energy Poverty Advisory Hub provides guidance and planning actions within the wider energy transition, ensuring that social justice dimensions of energy poverty are addressed^2^. Many argue that energy poverty is a violation of basic universal rights, as minimum energy services are needed for people to live in safe, clean and healthy environments^3^. The European Commission acknowledges that the three main variables contributing to energy poverty are (1) low income, (2) high energy expenditure and (3) low energy efficiency of the home. However, there are wider variables influencing energy poverty beyond the homes’ agency. For example, low income is influenced by job markets, energy expenditure by energy prices and homes’ efficiency by cold climate^4^. There have been growing calls for the EU to act on energy poverty^5,6^. However, there is insufficient research investigating how these three main variables can be targeted within a framework of energy justice involving: distributive (ensuring even distribution of benefits for all people), procedural (ensuring fair procedures and decision making for all people) and recognition justice (ensuring fair representation for all individuals)^7^. Recent research uses forecasting and modelling to show how certain policies within the EU can have negative or positive impacts for vulnerable groups in energy poverty^8,9^. Recent research^9^ finds that expenditure shares of residential energy declines with income, thus supporting operationalisation of affordability-based measures. They suggest that tailored policy interventions could raise 1.35 million EU homes out of energy poverty. The European Commission suggests indicators to measure energy poverty can vary depending on member-states but requires all countries which have a significant number of homes in energy poverty to formulate objectives to reduce it. To date, only 9 of the 32 member states have adopted an official energy poverty policy for it. The Netherlands is not one of them.

### Energy poverty in the Netherlands

Recent Dutch research^10^found that 550,000 homes, or 7% of the population in the Netherlands are classified as energy poor using the indicators of low income, low energy quality of homes and high energy bills. Low income is defined by the social minimum threshold from Statistics Netherlands (i.e. single person households = €1573). Low energy quality is defined by houses with energy label D or lower. High energy bills are defined as being above the national median. The Dutch government has a position but not a policy for energy poverty. This leaves municipalities, social welfare organizations and households with a lack of funding, framework and flexibility to assess and fix the problem. Dutch ministers suggest energy poverty is an economic problem and thus should be tackled through social welfare rather than energy policies^11^. Thus, energy poverty is targeted through a distributive justice lens only, primarily giving subsidies to those in need. However, experts suggest the issue requires changes not only in social welfare policy for low-income homes but in building policy for energy efficient homes and education policy for homes with low levels of energy education^11^.

### Energy poverty in Amsterdam

The renewable energy transition in Amsterdam is already underway, where it is expected that all homes should be natural gas free by 2050. Such transitions run the risk of exacerbating existing inequalities and further marginalising vulnerable parts of the population through increased costs and energy burdens^12^. But it also offers an opportunity for vulnerable homes to simultaneously reduce their energy related emissions and poverty. If governments first help homes manage more pressing energy concerns, such as energy poverty, which affects 9.3% of homes in Amsterdam, this may serve as a stepping stone to their participation in the wider energy transition^10^, thus addressing procedural justice. This requires guidance of the municipality Buurt-teams (who provide assistance for low-income homes), energy providers (who provide assistance with energy bills) and local social organizations, who provide assistance to help with the energy efficiency of the homes.

### Energy poverty interventions in the home

Global reviews of interventions in the home suggest that most use cross sectional design (only capturing data at single time points) or are quantitative only (not interviewing homes as to why changes occurred)^13^. We found 25 intervention studies explicitly targeting energy poverty, with positive results for secondary indicators such as increased mental wellbeing, increased perception of social support, improved energy literacy and thermal comfort^14–20^. However, 16 out of 25 interventions target efficiency measures only, while none target all three main variables (efficiency, income and consumption). Increasing the efficiency of the home is a first step to reducing the energy demand of the house. But homes still don’t have sufficient income to pay bills nor engage in energy efficient behaviours. Recent research considers energy limiting behaviours by income^21^, and suggests interventions include non energy-poor homes, so that energy equity gaps can be uncovered and address recognitional justice. Compared to efficiency interventions, little is known about the effects of information-based measures such as coaching. Energy coaching research^22^found psycho-social gains and the ability to keep the home warm after one year. However, this information was static and retroactive (i.e. annual bill) rather than dynamic and in real-time (i.e. smart devices). Smart technology policies are suggested^23^as they could empower energy poor homes to become more energy secure^6^but are rarely used to target energy poverty^9^, because they are not affordable for energy poor homes^24^.

### Research aims and approach

This study aims to fill gaps in energy poverty research by measuring (a) all three EU-wide variables: income, efficiency and consumption, (b) in the Netherlands where no policy exists, (c) in Amsterdam with local municipality services and (d) in homes using smart technology.

The intervention assessed the most widely and freely available energy coaching programme in Amsterdam, developed by a government housing organization. Participants were recruited when they contact to request help from an energy coach. The coach visits homes, asks energy questions and gives energy advice following a protocol, then installs energy efficient products and emails a report to the home a day later (Supplementary Material C). The lead author of this paper was trained by the government housing organization to become an energy coach and install energy efficient equipment. The researcher offered all homes smart energy devices which monitors real-time gas and electricity consumption in relation to their income. Fifty homes accepted the smart energy device and are in the smart information group, while sixty-seven did not and are in the static information group. The researcher visited 117 homes between October 1st and November 31st 2023, and revisited 73 of them between January 31st and March 31st 2024, to assess the observed effects and perceived affects of the intervention. We also found 52 control homes (with similar building stock/number of people per home in the same neighbourhoods), who received no energy coaching and their data was obtained after the heating season, therefore consumption was not influenced by the study. All steps are detailed in the method section.

This approach was used to address one quantitative and one qualitative question:

1) What are the quantitative effects of energy coaching in homes vulnerable to energy poverty over the heating season?

2) What are the qualitative affects of energy coaching in homes vulnerable to energy poverty over the heating season?

## Results

**Fig. 1:**
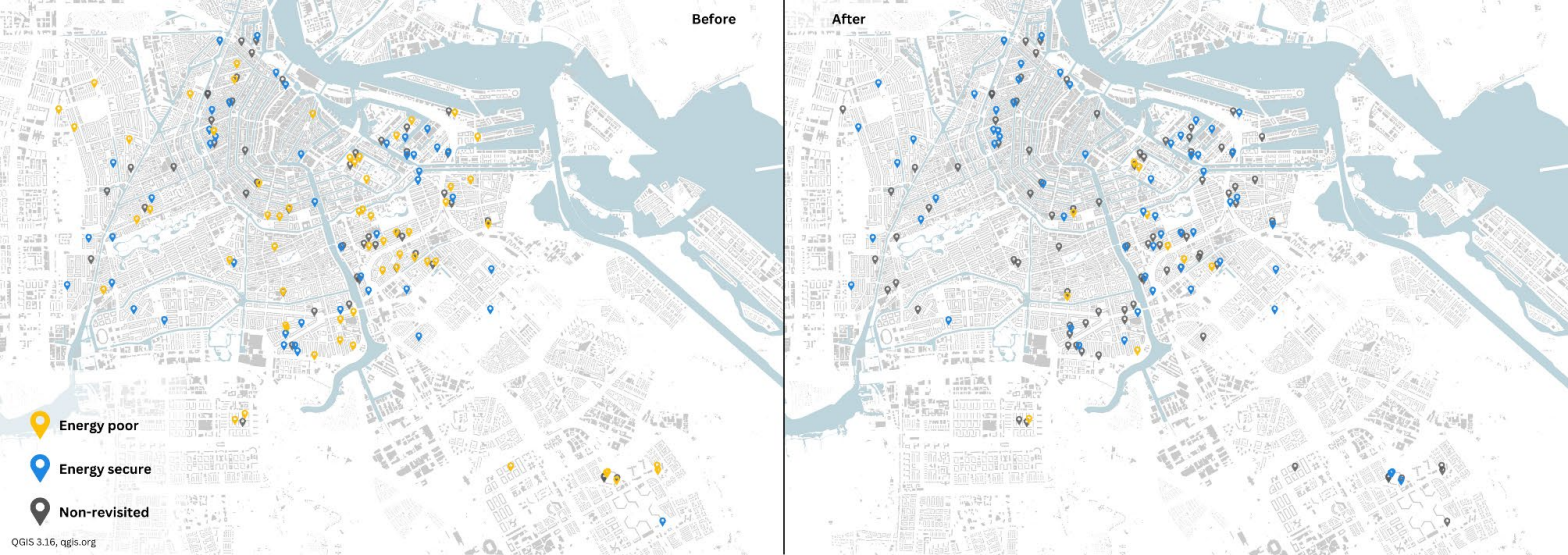
Baseline Homes (*N* = 117) (left image) & Revisited Homes (*N* = 73) (right image) in Amsterdam,.

Figure 1 was generated using open street map, credits go to all authors (available under the Open Database License, openstreetmap.org). Figure 1shows yellow icons are homes which spend more than 8% of income on energy (thus classified as being energy poor), whereas blue icons spend below 8%. This threshold aligns to Dutch national indicators to represent all homes^10^. It is calculated based on a standardized threshold of the social minimum and adjusted for financial assets beyond their income. In the baseline sample, 65 out of 117 homes were classified as being energy poor, with 44 on social assistance and 29 with high energy costs of +€236 per month based on national statistics^25^. 40 out of 73 revisited homes were energy poor pre-intervention (using the 8% indicator). 10 out of 73 homes remained energy poor post- intervention. All 10 of these homes were on social allowance and/or spent more than 20% of their income on energy to begin. All primary and secondary building/social indicators are in supplementary material A. All data generated during this study are included in this published article and its supplementary files.

### Efficiency results

**Fig. 2:**
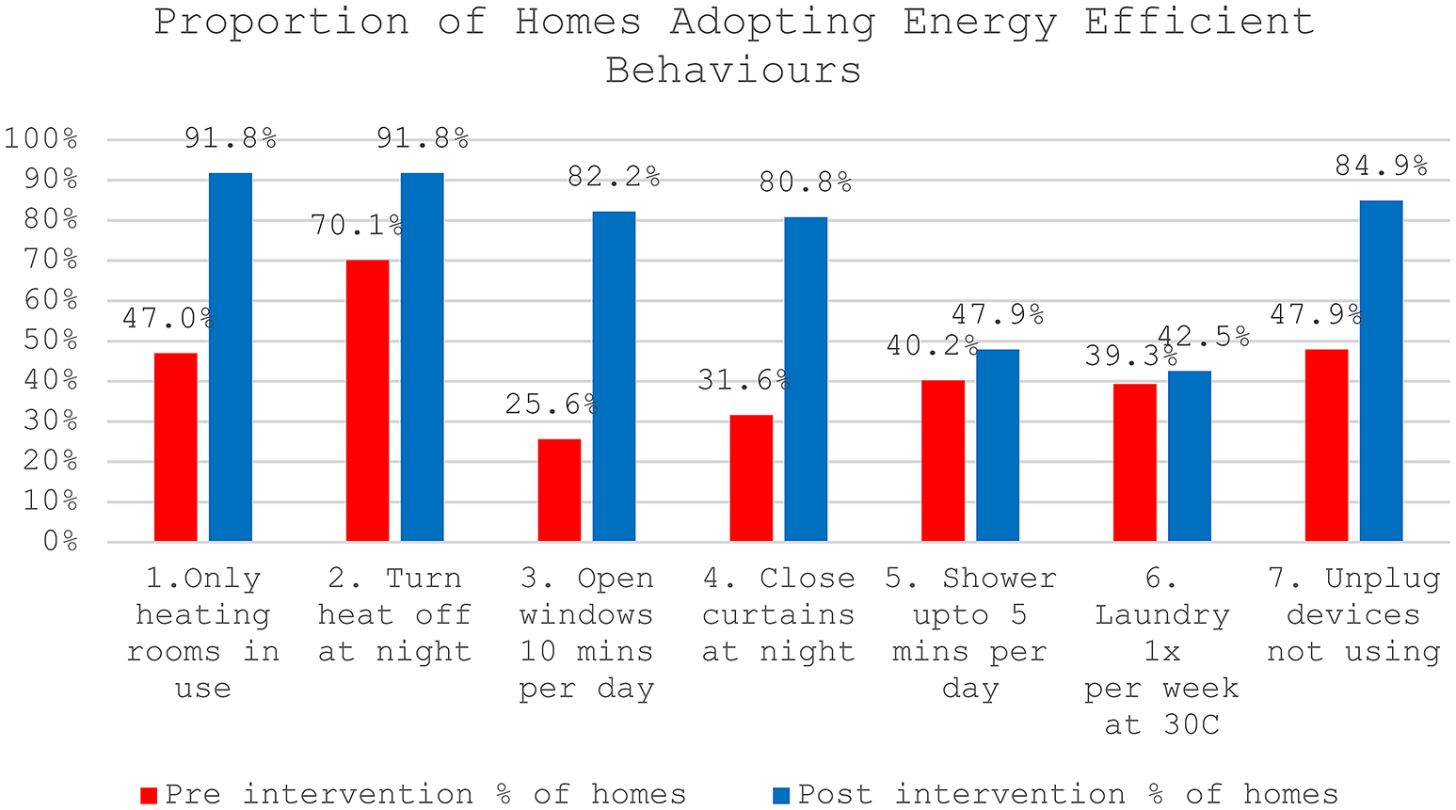
Pre/Post-intervention behaviour change across all smart and static information homes.

Findings from survey questions highlighted in Fig. 2 show the observed behavioural effects of the intervention, thus answering research question 1. Due to involving both energy poor and non-energy poor homes, the data was not normally distributed. Therefore, a non-parametric McNemar test found significant changes in proportions of homes who changed behaviours from pre to post-intervention, x^2^ (1, *n*= 73) for behaviour: 1. only heating rooms in use = 36.03, p = < 0.001, 3. opening windows 10 min per day = 36.31, p = > 0.0015, 4. closing curtains at night = 34.03, p = < .001and 7. unplugging devices when not in use = 25.04, p = > 0.001. Other behaviours (2, 5 and 6) positively changed but not significantly. When looking closer at the differences within static information homes only, we find that 4 out of 7 behaviours (all heating related) significantly changed from pre to post-intervention x^2^ (1, *n* = 31), including only heating rooms in use = 13.07, p = > 0.001, turning heating off at night = 7.11, *p* = .004, closing curtains at night = 8.11, *p*= .002 and opening windows 10 min per day = 13.07, p = > 0.001. Meanwhile, smart information homes significantly changed 6 out of 7 behaviours x^2^ (1, *n* = 42), except doing laundry = 0.5, *p* = .48. Thus, results suggest energy coaching can help homes adopt energy efficient behaviours, especially concerning heating. However, when paired with smart devices homes adopt more energy efficient behaviours.

### Consumption results

**Fig. 3:**
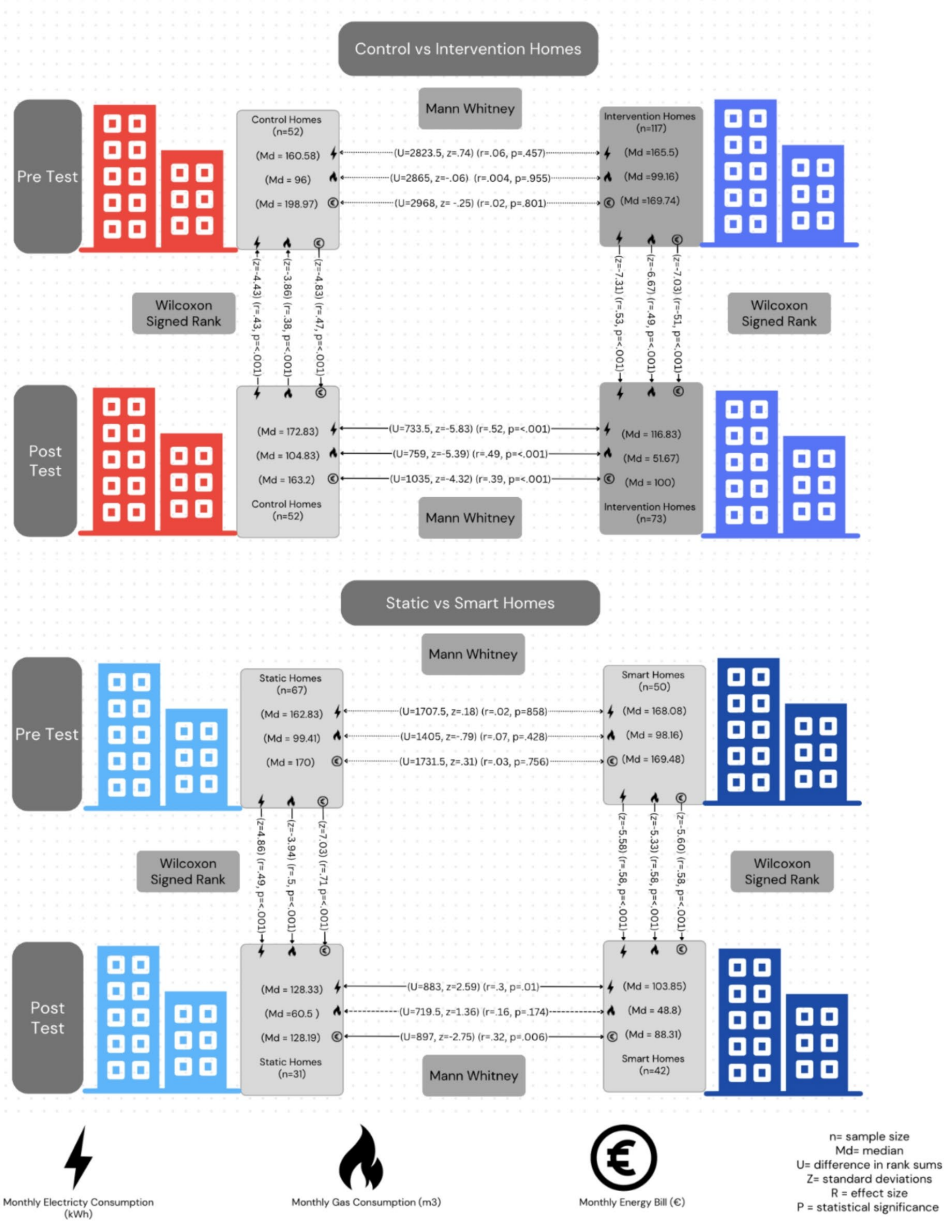
Statistical tests using Mann Whitney to determine significance between groups and Wicoxon Signed Rank to for significance within groups for monthly electricity, gas and energy bills.

Consumption data was collected via smart devices for the smart information group and via physical energy bills for static information and control groups. Data was aggregated to the monthly level to fairly compare across groups, as there is a 6-week gap between the first and last home visited. However, an equal number of smart and static information homes were visited each week during this time. Other uncertainties surrounding energy prices and outside weather were also controlled for as co-variates in post-hoc analysis (in the Method). A non-parametric Kruskal-Wallis test compared consumption across control, static and smart information homes, pre intervention to post intervention. There are no significant differences between the 3 groups for pre-intervention electricity (control *n* = 52, static information *n* = 67, smart information *n*= 50), x^2^ (2, *n* = 169) = 0.558, *p* = .756 or gas (control *n* = 51, static information *n* = 67, smart information *n* = 46), x2 (2, *n* = 164) = 0.628, *p* = .731. As shown in Fig. 3, a Mann-Whitney test found significant differences, where post electricity and gas consumption were significantly lower for all intervention homes compared to control homes. The Kruskal Wallis-test shows a significant effect across all three groups but does not show significant differences between specific groups. Therefore a Mann-Whitney test was performed in post-hoc, to identify significant differences between static vs. smart information homes. Here we find that post-intervention electricity consumption is higher in static information, than smart information homes and we find no significant difference for post-intervention gas consumption. Thus, the smart information is more effective at reducing electricity consumption than gas.

**Fig. 4:**
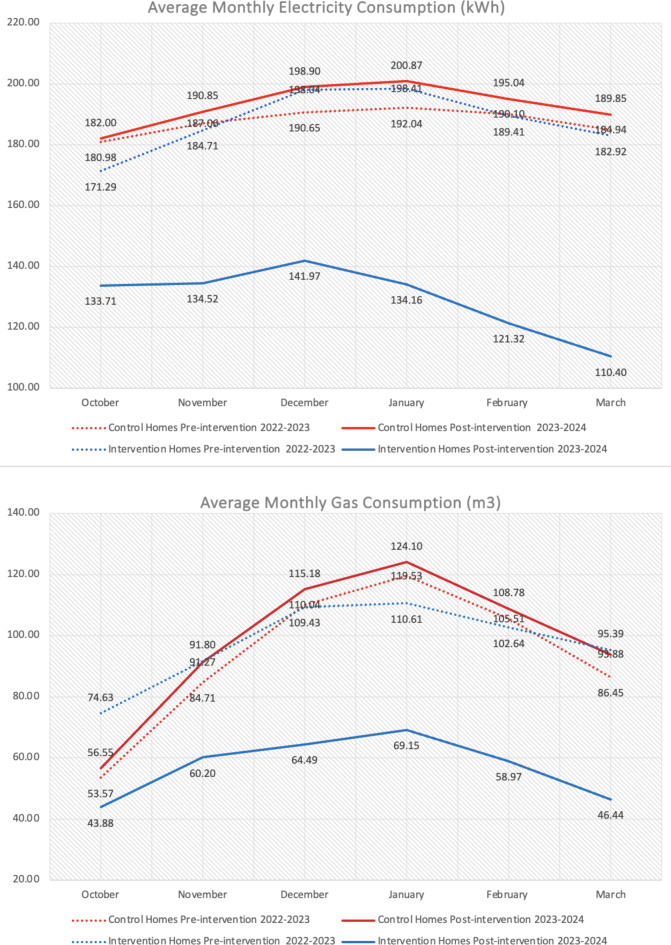
Pre/post-intervention mean monthly gas and electricity consumption between control and intervention homes across the heating season.

**Fig. 5:**
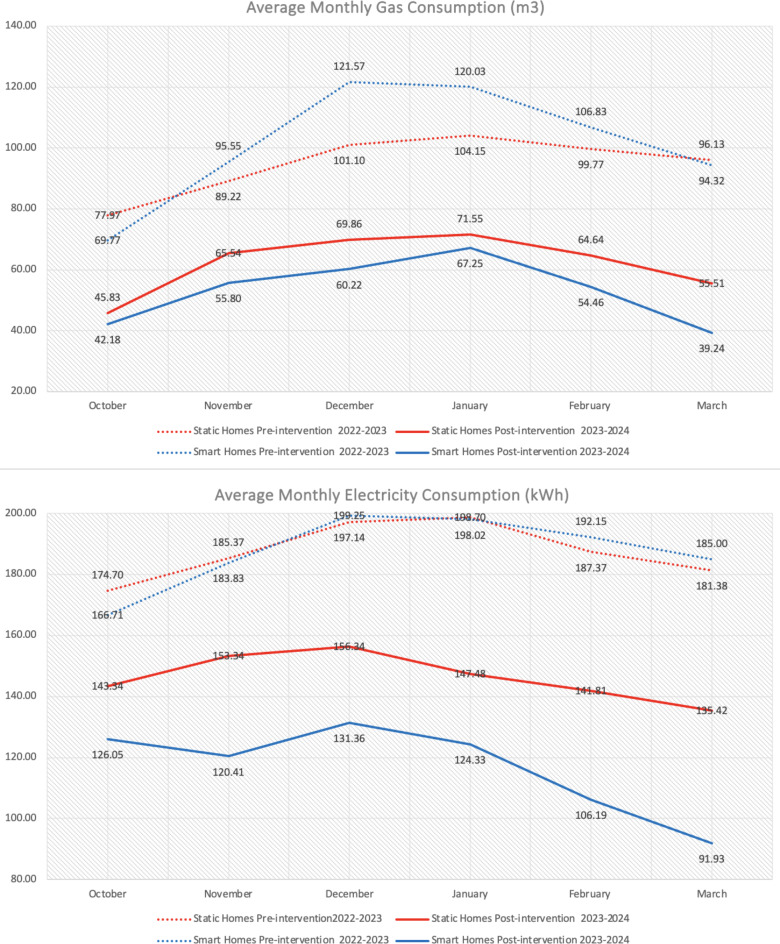
Pre/post-intervention mean monthly gas and electricity consumption between static and smart information homes across the heating season.

Next, a Wilcoxon Signed Rank test found significant differences in static homes electricity and gas consumption decreasing from pre to post intervention. Meanwhile greater decreases were found in smart homes electricity and gas consumption from pre to post intervention. As shown in Figs. 4 and 5, electricity and gas reduced within static and smart information homes but increased within control homes from pre to post intervention.

### Income results

**Fig. 6:**
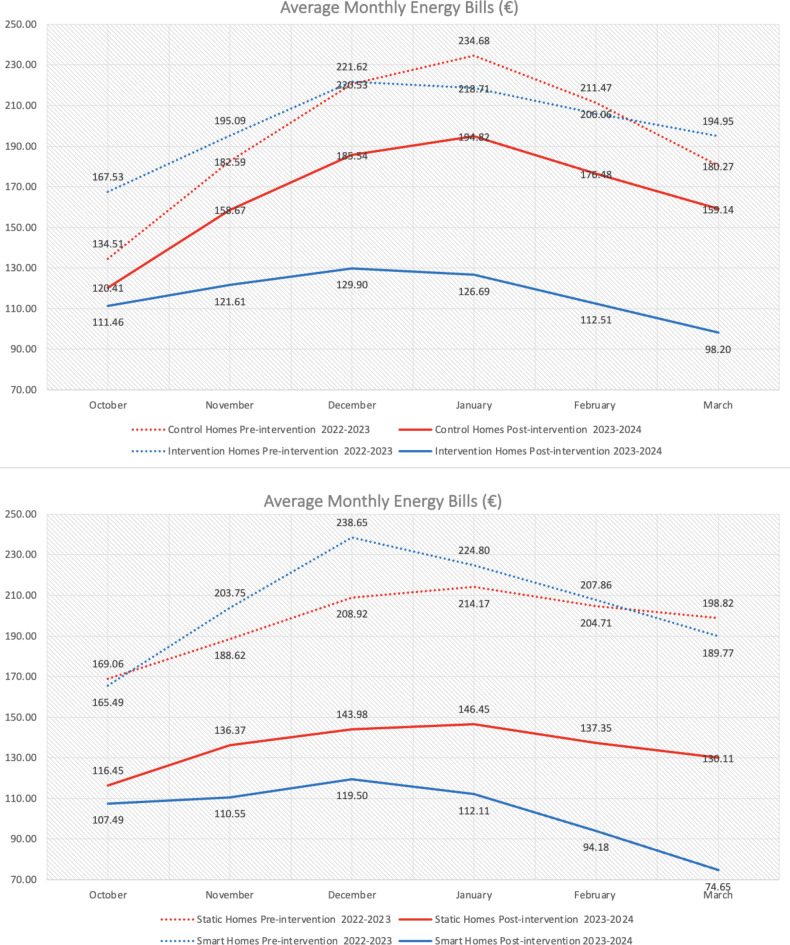
Pre/post intervention mean monthly energy bills between control and intervention homes then between static and smart information homes.

**Fig. 7:**
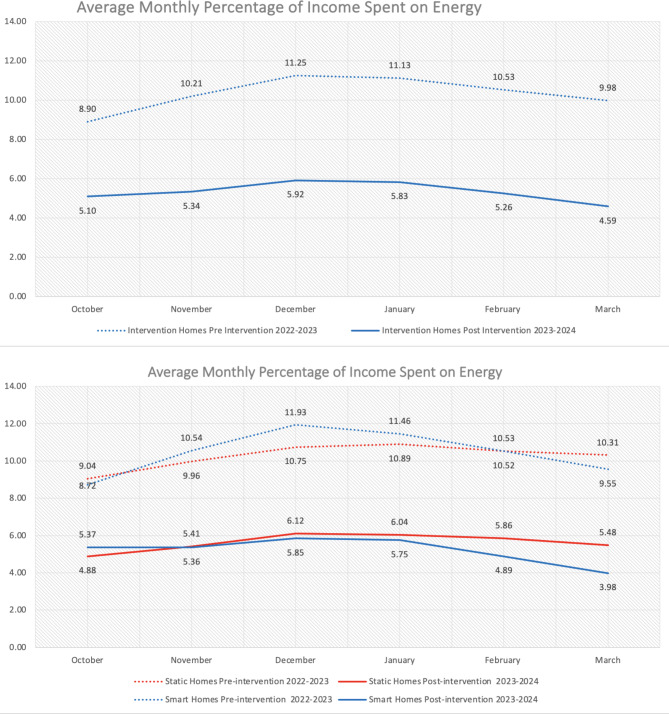
Pre-post intervention mean percentage of monthly income spent on energy first across all intervention homes then between static and smart information homes.

Income data was obtained via physical income statements from smart and static information homes. Only one homes’ income changed during the intervention (as they added an extra person to the household). However, no income data was obtained for the control group. A Kruskal-Wallis test shows no differences between the three groups for pre-intervention energy bills (control *n* = 52, static information *n* = 67, smart information *n*= 50), x^2^ (2, *n* = 169) = 0.119, *p* = .942. However, a Mann Whitney test found significant differences where post-intervention energy bills were higher for control than intervention homes. Next, we find a significant difference with post-intervention energy bills lower in smart than static homes. Furthermore, a Wilcoxon Signed Rank found significant differences in static homes energy bills decreasing from pre-intervention to post intervention. Yet, a greater difference was found in smart homes. Finally, when comparing percentage of monthly income spent on energy, intervention homes significantly decreased, but with greater decreases for smart than static homes. Thus, Figs. 6 and 7 show the smart intervention is more effective at reducing energy poverty.

### Interview results

Interviews from 73 homes highlight perceived affects of the intervention, answering RQ 2. Main motivations to engage in the project concerned saving money (“Yesterday we changed from ‘energy company X’ because it is too much. €220 is okay but I cannot eat with an energy bill of €305. Together with the food bank though it helps. I want less energy use, maybe €100 a month to help me. This is my aim!”, Mahira). When asked how and why they changed behaviours, participants suggest that heating behaviours were easy to change (“I’ve been smarter with using the heating. “I change the gas schedule and heating parameters. Before I was using automatic heating. Now I only heat when feeling cold”, Evie). Meanwhile, opening of windows was the behaviour that changed the most (“The house warms up quicker; it goes against your instinct. But it’s funny that it works. That helps our health a lot”, Tim & Fiona) When asked about the experience with the device, responses suggested it was more beneficial than coaching alone, “The coaching was interesting as we changed a lot. But with the device feedback we changed even more.” (Jitan & Sven) and that it helped them feel in control over their usage (“Some things don’t use as much energy as I thought. For a long time I didn’t feel like I had a grip on the usage but the thingy has helped”, Fleur) and (“I pay attention now to what I’m using. I hope at the end of this year I don’t get a bill as high as Mount Everest. The device is always green which is good”, Joy). When asked why the device was more effective for electricity than gas, residents pointed to housing problems that couldn’t be helped by the coaching, (“The problem is with him (points to thermostat) we need to put him to 22 C to feel anything at all”, Myra). When asked about the experience overall, residents appreciate the participatory nature of the coaching, suggesting procedural dimensions of energy poverty was addressed (“It’s very helpful that someone else thinks along with you. These are the types of things that usually fall last on your list”, Margaret).

## Discussion

### Energy Poverty Changes in the Home

Results show that energy coaching is effective at increasing energy efficient behaviours, reducing energy consumption and ultimately the percentage of income spent on energy, but even more so when homes received smart information. Previous smart technology interventions rarely targeted energy poverty^26^but those that did assuage the need for informational feedback to promote energy literacy^22,24^. Findings here suggest that compared to monthly energy bills, the energy device made them feel in control over their energy needs. *“If you have something real-time like this*,* you can calculate the bill so it is positive and makes you spend in a comfortable way. Control is everything*”, Ben). Previous energy poverty intervention reviews across the world show significant improvements in various mental health indicators^27^. By contrast our own interview findings suggest that feelings of being in control of energy needs improved for most homes. However, the device was sometimes described as ‘confrontational’, especially when red “*I’m constantly going to see the device but I’m getting stressed by it”*, Lucinda). It should not be the intention of any intervention to add stress to a home that is already under pressure to pay energy bills. Nevertheless, participants suggested that real-time information was effective to educate all family members at different times of the day (“The meter was in the central place in the house. Everyday we were watching it. Before you came the temperature was 20 now it is 18, at night it used to be 19 now it is 17. What surprised me was the simple solution changed our habits, it could be helpful if it was in every house to save energy nation-wide”, Davide).

### Energy Poverty Changes in Amsterdam

Participants here suggest the energy coaching project was helpful but there is much more municipality decision-makers can do to alleviate energy poverty in the city, (“*I can’t apply for funding. The government is a little passive versus active in their approach*”, Alix). 54 out of 65 energy poor are in homes labelled D or lower and 42 are in social housing. Mandatory landlord efficiency upgrades are policy suggestions for EU cities^6^. However, homes here suggest there is no will from social housing owners to upgrade homes to a standard of living that does not perpetuate energy poverty “*The social housing gave me a bill for €1700 which made me reconsider them. My son has bronchitis*,* the house has mould. I’ve been on the social housing for 12 years and hoping they finally renovate but they’ve not made things better here*”, Sandy). Energy poor homes in our sample were often unemployed, on welfare, older aged or with underlying health issues, thus spending more time at home which perpetuated energy poverty. A meta-analysis of energy efficiency interventions across the world finds significant improvements in participants physical health, when buildings are retrofitted^28^. Unfortunately, our intervention did not include deeper retrofits but with mould, damp or leaks being found in 65 of the 117 homes, our findings suggest these larger scale interventions are vital. Prior Dutch research^10^ found that 48% of homes in the Netherlands cannot participate in the energy transition because residents don’t own homes or have sufficient financial wealth. However, we also find that 45% of homes who received energy coaching, subsequently engaged in the energy transition in some small way. This ranged from applying for solar panel subsidies, securing retrofits from landlords, adding insulation, switching to renewable energy suppliers and replacing gas boilers with electric ones. This suggests energy coaching could be a procedural justice intervention to meaningfully engage homes in the city’s wider energy transition.

### Energy poverty changes in the Netherlands

While the Netherlands has no energy poverty policy, it has the lowest proportion of homes in debt on energy bills (1.1%) across all EU countries^2^. 4% of homes in our sample were in debt. This is still relatively low, where homes report that energy subsidies helped to cover costs. However, the Dutch central planning office^29^ suggests homes living below the poverty line increased from 815,000 to 995,000 in 2024. This is due to temporary welfare measures (i.e. energy subsidies) disappearing. Without previous subsidies, homes that are currently just below the 8% energy threshold are worried they will be on the wrong side of the line next year (“*My tariff I expect to increase which is worrisome it is making me stressed facing higher bills*”, Mario). Income based interventions are still vital^9^, especially as they address distributive economic justice. Prior energy poverty reviews around the world suggest interventions can effectively reduce the number of homes in arrears on energy bills^13^, however they do not detail the actual amounts homes have saved. By contrast, our smart devices have helped to calculate that on average homes in the smart information groups saved €104 per month over the heating season. Findings here can help policy by monitoring indicators from multiple sources, from national energy companies monitoring low prices, social housing providers monitoring energy label requirements, to local municipalities monitoring low-income needs, to research institutions monitoring frameworks and most importantly from homes here. Therefore, the Netherlands can address the whole problem, rather than only focus on income and distributive justice.

### Energy poverty changes in the EU

This study shows that all 3 main EU wide variables are relevant for reducing energy poverty. However, secondary indicators should still be acknowledged. All 65 energy poor homes in our sample were either on single income or social allowance, 22 of these had underlying health issues, 22 were ethnic minorities and single parent families were the most disproportionately affected (10 out of 12 were energy poor). All participants suggest that energy subsidies in 2022 and 2023 helped but did not help lift homes out of poverty in the long-term (“I want to feel comfortable but I just wont be able to pay it”, Julie). Unstable energy markets have exacerbated the issue, where a 4.8% increase in household expenditures is expected in the coming years for 116 countries around the world^30^. By contrast, our results show that both intervention groups reduced their percentage of income from 10.1% to 5.3%. No matter what threshold a country decides to use to measure energy poverty, an intervention which nearly halves their expenditures should be considered in policy making. Recent modelling research^9^argue for policy packages to be heterogenous to achieve public support. However, they suggest that a package which directly affects all attributes of energy poverty is difficult to deliver. Nevertheless, common provisions for basic universal rights and minimum protection against energy poverty, which recognises the 3 main factors can help EU policymakers^5^. EU packages can give each member state the money to meet the needs of all homes, national governments the autonomy to adapt policies with multiple actors, so local municipalities can implement measures which match heterogenous needs of homes, from social housing to single parent families. Therefore, including experiences of marginalised groups can address recognition justice too.

### Limitations and future directions

Our findings corroborates previous policy research stating that energy poverty is substantially an affordability issue^9^ and we argue that percentage of income spent is still the most useful. Findings here show this indicator aligns to primary motivations for most homes (to save money on bills). While none of the energy-poor homes described themselves as ‘energy poor’, they all shared concern about paying bills. However, energy poverty experiences encompass more than 8% of income spent on energy. Just because we define a home as energy poor, does not mean they experience themselves to be. For example, one ‘energy poor’ home increased their spending but described it as a positive experience. *“I love the meter of course. I wish we had done it sooner. Some things don’t use as much energy as I thought I’m not as scared anymore to put on the heater. That is the main difference. But I’m at home a little more and not freezing all the time. It is nice to watch the smart meter and be calm about it", Dorothea)”.* Her heating needs were now met with the help of the smart device. However, she was not the only home under consuming energy. The coaching advice given always emphasized a ‘healthy’ amount of energy-use for each home’s needs. In the Netherlands, under consuming homes include low-income homes, living in low energy quality homes with energy costs below the 25th percentile^10^. Data from our sample suggested that 24 homes were under consuming. However, homes here stated while they were content with their consumption they were not with bills. For Dutch context, they often described themselves as ‘zuinig’ which translates to frugal, economical or even just efficient with money.

Still, a key limitation of the study is the sample being dependent on homes contacting first. This means that truly vulnerable homes may be missing. All homes that took part were to some degree, motivated to reduce their bills. However, homes that may be living in worse housing conditions will feel less motivated to participate, due to stigma or social isolation. Therefore, research investigating end user requirements in the decision-making phase^31^is fruitful for targeted interventions to prevent regular poverty spiralling into energy poverty. A recognitional justice perspective of how energy poverty affects vulnerable social groups means policies are more effective^32^. Future research must investigate secondary indicators such as how social characteristics identified here (i.e. single parents) intersect with wider political landscapes they inhabit (i.e. a socially just energy transition).

## Conclusion

Quantitative findings here show that energy coaching is effective at increasing the energy efficiency of the home, reducing consumption and ultimately, the percentage of income spent on energy. For efficiency, the coaching helped homes adopt energy efficient behaviours, especially around heating, giving homes agency to determine the energy efficiency within the home. However, qualitative findings from interviews suggest that wider building structures and actors such as social housing owners can help or hinder efficiency to a greater extent than individuals alone. For consumption, findings show that both gas and electricity can be significantly reduced with energy coaching, especially when complimented with smart information. However, interviews suggest that for some homes to heat to a healthy standard, this requires increased consumption and a recognition of differences between how we define energy poverty and how it is experienced. For income, findings show that energy coaching can have a significant impact on income spent on energy, thus helping to alleviate the financial burden of energy poverty. While this frees up disposable income for other basic human rights (i.e. food, housing, healthcare and childcare) most homes are still on social assistance and therefore still living in poverty. An energy poverty policy which targets all main variables is required. This means simultaneously addressing building policy (ensuring houses meet basic living standards and incentivised social housing owners), education policy (ensuring homes have real-time information on their energy-use to take ownership and action) and social welfare policy (ensuring single income homes have enough basic income in the first place, so they can afford to meet these basic needs). No single intervention nor policy will effectively target the heart of the problem. However, actions here that engage homes needs first and foremost can provide a just and fair start.

## Methods

### Research design, intervention, participants, procedure and ethics.

An experimental intervention was conducted to study the effect of energy coaching (as the independent variable) on energy poverty (dependent variable). Baseline measurements were taken between October 2023 and December 2023, where 117 homes were visited. All homes were offered the chance of a revisit but due to scheduling conflicts and life circumstances only 73 were revisited to measure the impact of the intervention from February 2024 to March 2024. Opportunity sampling occurred, where homes would voluntarily reach out to the energy coach team and request a visit. The project and products offered are free of charge, therefore open to homes of all income groups. As such, this means both energy-poor and non energy-poor homes are in the sample which also means that participants were potentially predisposed to energy efficiency improvements.

Previous intervention research has been criticised for treating vulnerable actors as ‘passive receivers’^9^. Here the coaching involved learning about the home and installing the products together. The coaching usually focuses on saving energy. However, some homes may use so little (i.e. not turning their heating on at all during winter). For them, increasing energy efficiency, consumption and income they spend may be needed. Therefore, the coaching requires tailoring to circumstances and suggesting healthy energy-use rather than moralising about too much or too little energy consumption.

The coaching consisted of a 2 h session where energy related questions were asked, advice was given and energy efficient products were installed. Within one week of the visit, homes would also receive a tailored report giving savings and advice. Here, homes were offered the smart energy display, also free of charge. By giving homes the choice of which intervention group they wanted to be a part of (static or smart), we prioritised ethical concerns giving homes freedom of choice instead of prioritising a randomised control trial. Reasons for not installing the device included: concerns of data privacy, not wanting to be overloaded with information or simply not having access to their smart meter and not having one. Although having participants choose which group they want to be in can introduce self-selection bias which skews the results (i.e. those who are more tech-oriented would want the smart device). However, the socio-demographics across both samples are comparable (see supplementary material A). For example, both groups had on average 2 people per home, had between 20 and 28% pension aged people, 28–34% ethnic minorities, 40–50% social housing, and both samples had 70% of homes on single income. This suggests that self-selection may not bias results. Thus, we had 67 homes which only received static information (a single report) and 50 homes which received smart information (a smart display). The minimum sample size for a mixed-methods intervention has been suggested at *n*= 21 per group^33^. As our intervention groups have 67 and 50 respectively, we also found 52 homes (with similar building stock, number of people per home and in the same neighbourhoods) to serve as a control group comparing consumption across the intervention. Secondary indicators for measuring energy poverty were recorded, such as building characteristics (i.e. age and type) and social characteristics (i.e. health and gender). These are background characteristics, as participants could not make changes here.

## Efficiency

Home efficiency is largely determined by building characteristics, where prior research uses the energy label as a determinant of energy poverty^10^. While energy label data can be publicly sourced, the data set in previous research and many older homes in our sample is incomplete. Therefore, as we are investigating interventions which consider the agency of the person, a set of behavioural indicators for energy efficiency was used. As previous research has investigated energy behaviours on both sides of the spectrum, from energy limiting behaviours^21^to energy saving behaviours^34^ across 38 countries, we also use behavioural indicators. These are recorded by the survey with advice for homes which do not currently engage in said behaviours.

## Consumption

The consumption of the home is measured through energy bills and smart meter recordings. We acquired pre-intervention consumption data to compare the intervention period (October 2023 to March 2024) to the same period in the previous year. Gas and electricity costs were recorded with outdoor temperature, as these two confounding variables may be contextual conditions that effect consumption^10^. As shown in Figs. 8 and 9, the 2022–2023 average is 7.6 ^o^ C and 2023–2024 is 8.3 ^o^C. This 0.7 ^o^C difference is small but significant to make a difference in heating consumption. The average electricity costs for intervention homes were 0.53 €/kWh from 2022–2023 and 0.45€/kWh from 2023–2024. For gas costs this went from 1.18 €/m3 to 1.03 €/m3. Again, this. 08 €/kWh and 0.15€/m3 is small but significant to make a difference in energy consumption. Therefore, these were factored into a Quade’s test (non-parametric ANCOVA) but it did not find any significant effect of energy costs or outdoor temperature as possible covariates influencing consumption results. When controlling for electricity costs, the pre-intervention electric scores are still not significant between groups (Q = 2.70, df = 2, *p* = .071) and post-intervention electricity consumption is still significantly different between groups (Q = 27.04, df = 2, p = < 0.001). This was also true when controlling for gas costs on gas consumption with no significant difference between groups pre-intervention (Q = 1.24, df = 2, *p* = .292) but a difference between post-intervention groups (Q = 18.94, df = 2, p = < 0.001). As we measured during the heating season, we controlled for outdoor temperature on gas consumption, finding no significant difference between groups pre-intervention (Q = 0.43, df = 2, p = < 0.65) but significant differences post-intervention (Q = 20.87, df = 2, p = < 0.001).

**Table:** 

	October	November	December	January	February	March
2022–2023	14^o^ C	9 ^o^ C	4 ^o^ C	6 ^o^ C	6 ^o^ C	7 ^o^ C
2023–2024	13 ^o^ C	8 ^o^ C	8 ^o^ C	4 ^o^ C	8 ^o^ C	9 ^o^ C

(Source: Weather station from Schiphol Airport, World Meteorological Organization).

Figure 8. Average outdoor temperature in Amsterdam during the heating season 2022–2024.

**Table:** 

	October	November	December	January	February	March
Electricity 2022–2023	0.52 €/kWh	0.52 €/kWh	0.53 €/kWh	0.53 €/kWh	0.53 €/kWh	0.55 €/kWh
Electricity 2023–2024	0.45 €/kWh	0.46 €/kWh	0.45 €/kWh	0.45 €/kWh	0.45 €/kWh	0.45 €/kWh
Gas 2022–2023	1.14 €/m3	1.65 €/m3	1.78 €/m3	1.22 €/m3	1.21 €/m3	1.20 €/m3
Gas 2023–2024	1.02 €/m3	1.03€/m3	1.04 €/m3	1.03 €/m3	1.03 €/m3	1.03 €/m3

(Source: National Statistics Netherlands, CBS).

Figure 9. Average energy costs in Amsterdam during the heating season 2022–2024.

## Income

We used income statements given by homes, to assess whether they were low income, receiving social assistance, or spent more than 8% of their disposable income on energy. This included statements from 12 months before the intervention to the end of the intervention. 8% was chosen as an indicator, as it matched previous Dutch research^10^.

### Smart Information

The information received by the two intervention groups differed in one fundamental way. The static group received no extra feedback on their energy-use beyond the one emailed report, whereas the smart information group received real-time feedback on their gas (m^3^), electricity (kWh) and the costs (€/hour) too. We assumed that this smart device gets closer to an effective energy poverty intervention, as it gives homes real-time information on all 3 variables: income (how much € they are spending), consumption (how much m3 and kWh they are using) and efficiency (how much each behaviour influences the display).

## Qualitative affect

The indicators identified above we expected to change based on previous research. While they can inform us about what changed, they do not explain how and why these changes occurred (or not), both between and within homes. As such, repeat interviews included questions pertaining to the experience of the energy coaching, how and why each efficiency behaviour changed (or not), and their experience with the energy display.

## Electronic supplementary material

Below is the link to the electronic supplementary material.

Supplementary Material 1

Supplementary Material 2

Supplementary Material 3

## Data Availability

All data generated during this study are included in this published article and its supplementary files.

## References

[1] Boeri, A., Gianfrate, V., Boulanger, S. O. M. & Massari, M. Future design approaches for energy poverty: users profiling and services for no-vulnerable condition. *Energies* **13** (8), 2115 (2020).

[2] European Commission. / Energy poverty indicators. Retrieved from (2023). https://energy-poverty.ec.europa.eu/epah-indicators

[3] Sovacool, B. K., Heffron, R. J., McCauley, D. & Goldthau, A. Energy decisions reframed as justice and ethical concerns. *Nat. Energy*. **1** (5), 1–6 (2016).

[4] European Commission, Joint Research Centre. Who’s energy poor in the EU? It’s more complex than it seems. *Joint Research Centre*. (2024)., September 25 https://joint-research-centre.ec.europa.eu/jrc-news-and-updates/whos-energy-poor-eu-its-more-complex-it-seems-2024-09-25_en

[5] Dobbins, A., Fuso Nerini, F., Deane, P. & Pye, S. Strengthening the EU response to energy poverty. *Nat. Energy*. **4** (1), 2–5 (2019).

[6] Sovacool, B. K. et al. Policy prescriptions to address energy and transport poverty in the United Kingdom. *Nat. Energy*. **8** (3), 273–283 (2023).

[7] Jenkins, K., McCauley, D., Heffron, R., Stephan, H. & Rehner, R. Energy justice: a conceptual review. *Energy Res. Social Sci.* **11**, 174–182 (2016).

[8] González Garibay, M., Primc, K. & Slabe-Erker, R. Insights into advanced models for energy poverty forecasting. *Nat. Energy*. **8** (9), 903–905 (2023).

[9] Vandyck, T., Della Valle, N., Temursho, U. & Weitzel, M. EU climate action through an energy poverty lens. *Sci. Rep.* **13** (1), 6040 (2023). 37055454 10.1038/s41598-023-32705-2 PMC10102115

[10] Mulder, P., Dalla Longa, F. & Straver, K. Energy poverty in the Netherlands at the national and local level: a multi-dimensional spatial analysis. *Energy Res. Social Sci.* **96**, 102892 (2023).

[11] Feenstra, M., Middlemiss, L., Hesselman, M., Straver, K., Herrero, T. & S Humanising the energy transition: towards a national policy on energy poverty in the Netherlands. *Front. Sustainable Cities*. **3**, 645624 (2021).

[12] Wang, Q. et al. Examining energy inequality under the rapid residential energy transition in China through household surveys. *Nat. Energy*. **8** (3), 251–263 (2023).

[13] Ballesteros-Arjona, V. et al. What are the effects of energy poverty and interventions to ameliorate it on people’s health and well-being? A scoping review with an equity lens. *Energy Res. Social Sci.* **87**, 102456 (2022).

[14] Lorenc, A. et al. Tackling fuel poverty through facilitating energy tariff switching: a participatory action research study in vulnerable groups. *Public. Health*. **127** (10), 894–901 (2013). 24120311 10.1016/j.puhe.2013.07.004

[15] Doll, S. C., Davison, E. L. & Painting, B. R. Weatherization impacts and baseline indoor environmental quality in low income single-family homes. *Build. Environ.* **107**, 181–190 (2016).

[16] Grey, C. N., Schmieder-Gaite, T., Jiang, S., Nascimento, C. & Poortinga, W. Cold homes, fuel poverty and energy efficiency improvements: a longitudinal focus group approach. *Indoor Built Environ.* **26** (7), 902–913 (2017). 28890663 10.1177/1420326X17703450 PMC5571750

[17] Peralta, A. et al. Impact of energy efficiency interventions in public housing buildings on cold-related mortality: a case-crossover analysis. *Int. J. Epidemiol.* **46** (4), 1192–1201 (2017). 28052930 10.1093/ije/dyw335

[18] Bhushan, N., Steg, L. & Albers, C. Studying the effects of intervention programmes on household energy saving behaviours using graphical causal models. *Energy Res. Social Sci.* **45**, 75–80 (2018).

[19] Vurro, G., Santamaria, V., Chiarantoni, C. & Fiorito, F. Climate Change Impact on Energy Poverty and Energy Efficiency in the Public Housing Building Stock of Bari, Italy. *Climate* **10** (4), 55 (2022).

[20] Simshauser, P. The 2022 energy crisis: fuel poverty and the impact of policy interventions in Australia’s National Electricity Market. *Energy Econ.* **121**, 106660 (2023).

[21] Cong, S., Nock, D., Qiu, Y. L. & Xing, B. Unveiling hidden energy poverty using the energy equity gap. *Nat. Commun.* **13** (1), 2456 (2022). 35508551 10.1038/s41467-022-30146-5 PMC9068781

[22] Carrere, J. et al. Effectiveness of an energy-counseling intervention in reducing Energy Poverty: evidence from a quasi-experimental study in a southern European City. *J. Urb. Health*. **99** (3), 549–561 (2022). 10.1007/s11524-022-00642-6 PMC9187783 35622196

[23] Sareen, S. Digitalisation and social inclusion in multi-scalar smart energy transitions. *Energy Res. Social Sci.* **81**, 102251 (2021).

[24] Caballero, N. & Della Valle, N. Tackling energy poverty through behavioral change: a pilot study on social comparison interventions in social housing districts. *Front. Sustainable Cities*. **2**, 601095 (2021).

[25] Nibud Kosten van energie en water. *Nibud*. Retrieved February 20, 2024, from (2024)., January 29 https://www.nibud.nl/onderwerpen/uitgaven/kosten-energie-water/#Gasverbruik

[26] Iweka, O., Liu, S., Shukla, A. & Yan, D. Energy and behaviour at home: a review of intervention methods and practices. *Energy Res. Social Sci.* **57**, 101238 (2019).

[27] Middlemiss, L., Stevens, M., Ambrosio-Albalá, P., Pellicer-Sifres, V. & van Grieken, A. How do interventions for energy poverty and health work? *Energy Policy*. **180**, 113684 (2023).

[28] Maidment, C. D., Jones, C. R., Webb, T. L., Hathway, E. A. & Gilbertson, J. M. The impact of household energy efficiency measures on health: a meta-analysis. *Energy Policy*. **65**, 583–593 (2014).

[29] CPB Netherlands Bureau for Economic Policy Analysis. Centraal economisch plan (CEP) 2023. Retrieved from (2023). https://www.cpb.nl/centraal-economisch-plan-cep-2023

[30] *Nature Energy*, *8*(3), 304–316.

[31] Abbasi, M. H. et al. Planning energy interventions in buildings and tackling fuel poverty: can two birds be fed with one scone? *Energy Res. Social Sci.* **93**, 102841 (2022).

[32] Jacques-Aviñó, C. et al. Qualitative evaluation of an intervention to reduce energy poverty: effects perceived by participants according to typologies of social vulnerability. *Energy Policy*. **167**, 113006 (2022).

[33] Onwuegbuzie, A. J. & Collins, K. M. A typology of mixed methods sampling designs in social science research. *Qualitative Rep.* **12** (2), 281–316 (2007).

[34] Piao, X. & Managi, S. Household energy-saving behavior, its consumption, and life satisfaction in 37 countries. *Sci. Rep.* **13** (1), 1382 (2023). 36697440 10.1038/s41598-023-28368-8 PMC9876990

