# Supplementary material for: Assessing the impact of energy coaching with smart technology interventions to alleviate energy poverty
Source: Sci Rep. 2025 Jan 13;15:969. doi: 10.1038/s41598-024-80773-9 (PMC11730312; doi:10.1038/s41598-024-80773-9)
Supplement: Supplementary file 1 — Supplementary Material 1 [file 41598_2024_80773_MOESM1_ESM.docx]

Supplementary Material A. Energy Poverty Intervention Indicators across all Homes

| **Groups** | **Pre-test Energy Efficient Behaviours Adopted** | **Post-test Energy Efficient Behaviours Adopted** | **Pre-test Average Monthly Electric Consumption (kWh)** | **Post-test Average Monthly Electric Consumption (kWh)** | **Pre-test Average Monthly Gas Consumption (m3)** | **Post-test Average Monthly Gas Consumption (m3)** | **Pre test Average Monthly Energy Bill (€)** | **Post test Average Monthly Energy Bill (4)** | **Pre test % Income Spent on Energy** | **Post test % Income Spent on Energy** |
| --- | --- | --- | --- | --- | --- | --- | --- | --- | --- | --- |
| Static | 6.00 | 7.00 | 95.67 | 68.50 | 98.00 | 60.50 | 188.83 | 115.13 | 12.59 | 7.68 |
| Static | 6.00 | 7.00 | 712.33 | 507.57 | 36.33 | 25.88 | 408.22 | 204.27 | 13.61 | 6.81 |
| Static | 0.00 | 4.00 | 300.00 | 264.17 | 108.33 | 61.33 | 256.67 | 191.53 | 17.11 | 12.77 |
| Static | 5.00 |  | 225.50 |  | 119.00 |  | 108.00 |  | 1.85 |  |
| Static | 3.00 | 4.00 | 333.33 | 308.67 | 8.33 | 7.17 | 124.17 | 112.69 | 2.89 | 2.62 |
| Static | 5.00 | 7.00 | 134.17 | 119.50 | 126.67 | 115.17 | 313.67 | 214.79 | 12.52 | 7.94 |
| Static | 4.00 | 6.00 | 140.17 | 108.17 | 14.33 | 9.33 | 189.70 | 143.80 | 9.48 | 7.19 |
| Static | 1.00 |  | 243.00 |  | 109.00 |  | 280.00 |  | 7.78 |  |
| Static | 4.00 | 7.00 | 88.42 | 79.83 | 52.66 | 27.00 | 155.11 | 71.08 | 3.30 | 1.51 |
| Static | 2.00 | 5.00 | 133.00 | 116.83 | 100.94 | 103.67 | 140.84 | 150.40 | 3.06 | 3.27 |
| Static | 5.00 | 7.00 | 190.00 | 155.00 | 102.50 | 60.00 | 209.25 | 140.60 | 7.47 | 5.02 |
| Static | 2.00 | 3.00 | 132.50 | 122.50 | 120.00 | 65.17 | 143.78 | 91.73 | 5.75 | 3.67 |
| Static | 3.00 | 6.00 | 190.00 | 160.00 | 121.16 | 80.00 | 93.00 | 93.00 | 5.17 | 5.17 |
| Static | 2.00 | 5.00 | 155.50 | 134.33 | 248.83 | 123.00 | 397.03 | 225.93 | 6.62 | 3.77 |
| Static | 4.00 | 5.00 | 208.83 | 199.83 | 158.50 | 145.83 | 323.05 | 303.00 | 9.79 | 9.18 |
| Static | 5.00 | 5.00 | 153.67 | 128.33 | 102.00 | 73.03 | 155.81 | 153.00 | 0.16 | 0.15 |
| Static | 3.00 | 3.00 | 188.00 | 160.00 | 100.00 | 65.00 | 150.00 | 150.00 | 10.00 | 10.00 |
| Static | 5.00 | 8.00 | 180.00 | 155.00 | 92.00 | 60.00 | 144.00 | 144.00 | 7.15 | 7.15 |
| Static | 3.00 | 6.00 | 160.33 | 159.80 | 79.50 | 37.67 | 144.60 | 64.42 | 2.48 | 1.10 |
| Static | 3.00 | 5.00 | 96.00 | 80.00 | 71.66 | 44.67 | 173.62 | 85.77 | 10.21 | 5.05 |
| Static | 5.00 |  | 195.00 |  | 100.00 |  | 170.00 |  | 0.16 |  |
| Static | 5.00 | 7.00 | 182.50 | 145.40 | 52.67 | 109.60 | 224.19 | 217.62 | 12.23 | 11.87 |
| Static | 6.00 | 7.00 | 109.00 | 90.80 | 30.00 | 28.00 | 116.23 | 74.96 | 10.57 | 6.81 |
| Static | 4.00 | 7.00 | 157.83 | 145.20 | 95.00 | 63.00 | 129.71 | 100.59 | 9.63 | 7.74 |
| Static | 6.00 | 7.00 | 144.67 | 123.80 | 51.33 | 48.20 | 144.85 | 128.19 | 5.79 | 5.13 |
| Static | 5.00 | 6.00 | 154.17 | 124.20 | 134.00 | 102.60 | 229.50 | 188.16 | 4.59 | 3.76 |
| Static | 2.00 | 4.00 | 103.00 | 92.00 | 136.00 | 86.80 | 282.56 | 124.38 | 21.74 | 9.57 |
| Static | 3.00 |  | 138.00 |  | 68.67 |  | 158.20 |  | 12.66 |  |
| Static | 2.00 | 6.00 | 153.83 | 145.20 | 93.20 | 66.40 | 135.96 | 90.59 | 11.52 | 7.68 |
| Static | 6.00 | 7.00 | 127.00 | 119.40 | 64.67 | 56.80 | 114.66 | 129.67 | 2.55 | 2.88 |
| Static | 4.00 | 5.00 | 140.00 | 134.40 | 30.00 | 18.60 | 100.00 | 100.00 | 6.67 | 6.67 |
| Static | 2.00 |  | 152.67 |  | 231.00 |  | 251.25 |  | 1.26 |  |
| Static | 4.00 |  | 162.00 |  | 97.33 |  | 300.83 |  | 3.01 |  |
| Static | 4.00 |  | 134.00 |  | 26.17 |  | 332.78 |  | 30.25 |  |
| Static | 5.00 | 7.00 | 101.50 | 80.75 | 53.00 | 43.75 | 128.71 | 80.02 | 8.58 | 5.33 |
| Static | 4.00 | 6.00 | 104.00 | 102.00 | 23.81 | 23.09 | 60.14 | 58.75 | 2.41 | 2.35 |
| Static | 3.00 | 7.00 | 140.33 | 139.00 | 31.50 | 17.00 | 128.43 | 67.58 | 10.70 | 5.63 |
| Static | 1.00 | 5.00 | 111.50 | 105.00 | 92.00 | 106.00 | 180.00 | 187.27 | 7.50 | 7.80 |
| Static | 5.00 |  | 200.00 |  | 105.00 |  | 311.00 |  | 33.26 |  |
| Static | 1.00 |  | 215.66 |  | 72.00 |  | 370.00 |  | 23.13 |  |
| Static | 1.00 |  | 228.33 |  | 75.00 |  | 100.00 |  | 2.00 |  |
| Static | 5.00 |  | 166.33 |  | 47.50 |  | 65.00 |  | 5.90 |  |
| Static | 5.00 |  | 132.16 |  | 124.66 |  | 156.00 |  | 10.40 |  |
| Static | 4.00 |  | 190.00 |  | 100.00 | . | 410.00 |  | 8.14 |  |
| Static | 6.00 |  | 183.33 |  | 166.67 |  | 300.00 |  | 20.00 |  |
| Static | 6.00 |  | 222.83 |  | 99.41 |  | 284.00 |  | 29.58 |  |
| Static | 4.00 |  | 110.66 |  | 62.66 |  | 60.00 |  | 2.88 |  |
| Static | 6.00 |  | 188.00 |  | 164.50 |  | 112.00 |  | 6.86 |  |
| Static | 5.00 |  | 298.91 |  | 84.33 |  | 259.00 |  | 3.24 |  |
| Static | 3.00 |  | 175.00 |  | 116.66 |  | 140.00 |  | 3.11 |  |
| Static | 7.00 |  | 208.33 |  | 72.66 |  | 59.00 |  | 2.74 |  |
| Static | 3.00 |  | 541.66 |  | 225.00 |  | 300.00 |  | 10.00 |  |
| Static | 5.00 |  | 195.00 |  | 95.00 |  | 172.00 |  | 10.12 |  |
| Static | 5.00 |  | 151.83 |  | 100.00 |  | 120.00 |  | 10.00 |  |
| Static | 4.00 |  | 227.00 |  | 103.83 |  | 150.00 |  | 6.00 |  |
| Static | 3.00 |  | 200.00 |  | 100.00 |  | 150.00 |  | 10.00 |  |
| Static | 3.00 |  | 333.33 |  | 129.16 |  | 190.00 |  | 9.50 |  |
| Static | 5.00 |  | 137.00 |  | 128.00 |  | 300.00 |  | 7.50 |  |
| Static | 4.00 |  | 118.66 |  | 101.66 |  | 150.00 |  | 10.00 |  |
| Static | 3.00 |  | 210.00 |  | 133.33 |  | 200.00 |  | 16.67 |  |
| Static | 1.00 |  | 220.00 |  | 120.00 |  | 300.00 |  | 34.88 |  |
| Static | 4.00 |  | 147.33 |  | 63.33 |  | 150.00 |  | 10.46 |  |
| Static | 8.00 |  | 162.83 |  | 29.33 |  | 325.00 |  | 10.83 |  |
| Static | 5.00 |  | 125.00 |  | 63.33 |  | 275.00 |  | 26.54 |  |
| Static | 2.00 |  | 190.00 |  | 82.00 |  | 190.00 |  | 12.67 |  |
| Static | 7.00 |  | 250.00 |  | 100.00 |  | 175.00 |  | 11.67 |  |
| Static | 6.00 |  | 260.00 |  | 110.00 |  | 165.00 |  | 11.00 |  |
| Smart | 6.00 | 7.00 | 286.00 | 157.00 | 108.00 | 64.50 | 100.40 | 90.43 | 2.01 | 1.81 |
| Smart | 4.00 | 8.00 | 109.50 | 70.00 | 108.00 | 50.00 | 97.80 | 63.00 | 3.26 | 2.10 |
| Smart | 2.00 | 8.00 | 585.36 | 109.83 | 109.83 | 56.00 | 274.46 | 103.60 | 20.79 | 7.85 |
| Smart | 3.00 | 6.00 | 237.50 | 73.83 | 99.16 | 48.17 | 264.12 | 85.60 | 22.97 | 7.44 |
| Smart | 3.00 | 6.00 | 101.00 | 107.00 | 131.50 | 130.50 | 164.11 | 165.84 | 10.94 | 0.06 |
| Smart | 3.00 | 6.00 | 193.83 | 102.17 | 241.33 | 100.50 | 258.53 | 140.73 | 13.61 | 7.41 |
| Smart | 5.00 | 6.00 | 76.00 | 45.50 | 83.17 | 42.87 | 85.76 | 49.37 | 3.43 | 1.97 |
| Smart | 4.00 | 6.00 | 276.33 | 81.33 | 130.33 | 37.50 | 302.32 | 97.23 | 21.86 | 7.03 |
| Smart | 2.00 | 5.00 | 170.67 | 127.00 | 111.50 | 109.33 | 216.86 | 150.67 | 3.61 | 2.51 |
| Smart | 1.00 | 5.00 | 136.67 | 84.50 |  |  | 88.83 | 50.70 | 8.88 | 5.07 |
| Smart | 2.00 | 5.00 | 184.50 | 99.50 | 85.00 | 2.23 | 194.58 | 78.63 | 18.53 | 7.49 |
| Smart | 4.00 | 8.00 | 268.17 | 81.30 | 53.17 | 4.25 | 150.89 | 66.28 | 13.72 | 6.03 |
| Smart | 3.00 | 6.00 | 135.17 | 62.00 | 112.50 | 52.33 | 83.89 | 37.19 | 1.68 | 0.74 |
| Smart | 4.00 | 8.00 | 89.50 | 64.00 | 105.17 | 39.50 | 130.02 | 54.18 | 5.20 | 2.17 |
| Smart | 2.00 | 7.00 | 228.67 | 105.50 | 231.50 | 109.00 | 336.86 | 162.82 | 28.07 | 13.57 |
| Smart | 3.00 | 7.00 | 229.00 | 164.00 | 132.92 | 80.00 | 201.16 | 79.28 | 14.37 | 5.66 |
| Smart | 2.00 | 7.00 | 184.50 | 120.80 | 97.17 | 61.00 | 127.73 | 82.16 | 1.16 | 0.75 |
| Smart | 7.00 | 7.00 | 163.67 | 136.33 | 51.17 | 29.83 | 116.23 | 82.12 | 8.94 | 6.32 |
| Smart | 4.00 | 7.00 | 85.17 | 58.17 | 78.67 | 55.50 | 260.78 | 101.42 | 10.43 | 4.06 |
| Smart | 5.00 | 8.00 | 157.50 | 88.50 | 125.33 | 49.33 | 169.21 | 87.69 | 4.57 | 2.37 |
| Smart | 5.00 | 6.00 | 422.00 | 119.33 | 140.00 | 81.83 | 392.90 | 171.63 | 41.40 | 18.09 |
| Smart | 4.00 | 6.00 | 108.00 | 98.33 | 49.50 | 42.50 | 72.45 | 64.17 | 5.57 | 4.94 |
| Smart | 3.00 |  | 155.25 |  | 100.25 |  | 291.50 |  | 7.29 |  |
| Smart | 4.00 | 6.00 | 160.17 | 102.20 | 97.00 | 48.80 | 231.41 | 93.84 | 8.26 | 3.35 |
| Smart | 6.00 | 7.00 | 56.50 | 49.60 | 22.17 | 19.60 | 72.11 | 47.76 | 8.01 | 5.31 |
| Smart | 3.00 | 6.00 | 100.17 | 71.60 | 82.42 | 40.50 | 120.84 | 68.33 | 3.02 | 1.71 |
| Smart | 4.00 | 7.00 | 179.17 | 132.60 | 114.25 | 62.00 | 225.33 | 121.47 | 4.51 | 2.43 |
| Smart | 3.00 | 7.00 | 125.83 | 93.20 | 91.33 | 38.00 | 207.20 | 66.10 | 13.81 | 4.41 |
| Smart | 3.00 | 6.00 | 118.00 | 132.00 | 53.17 | 69.20 | 169.74 | 172.94 | 8.10 | 8.25 |
| Smart | 6.00 | 7.00 | 395.00 | 225.80 | 92.50 | 38.40 | 670.63 | 249.86 | 26.83 | 9.99 |
| Smart | 2.00 | 6.00 | 89.00 | 62.20 | 82.17 | 30.20 | 109.82 | 50.85 | 3.66 | 1.70 |
| Smart | 2.00 | 5.00 | 398.00 | 270.60 |  |  | 136.18 | 92.86 | 4.13 | 2.81 |
| Smart | 3.00 | 6.00 | 69.83 | 53.80 | 69.50 | 11.82 | 124.48 | 42.59 | 11.32 | 3.87 |
| Smart | 1.00 | 6.00 | 491.67 | 299.00 |  |  | 491.67 | 204.44 | 10.24 | 4.26 |
| Smart | 4.00 | 7.00 | 148.33 | 126.80 | 55.50 | 31.30 | 349.38 | 165.36 | 9.71 | 4.59 |
| Smart | 4.00 | 7.00 | 235.33 | 109.60 | 109.50 | 51.00 | 330.08 | 115.24 | 5.50 | 1.92 |
| Smart | 3.00 | 6.00 | 196.83 | 148.00 | 136.83 | 59.20 | 119.59 | 62.16 | 1.20 | 0.62 |
| Smart | 5.00 | 7.00 | 198.33 | 108.25 | 95.00 | 36.25 | 201.46 | 88.93 | 16.79 | 7.41 |
| Smart | 1.00 |  | 92.43 |  | 124.79 |  | 161.94 |  | 5.40 |  |
| Smart | 3.00 | 8.00 | 165.50 | 114.25 | 89.83 | 91.00 | 322.51 | 141.25 | 8.06 | 3.53 |
| Smart | 5.00 | 8.00 | 98.83 | 78.00 | 65.33 | 17.50 | 115.91 | 26.69 | 8.28 | 1.91 |
| Smart | 3.00 | 7.00 | 117.67 | 57.00 | 84.00 | 20.25 | 121.33 | 45.29 | 1.21 | 0.45 |
| Smart | 4.00 | 6.00 | 248.00 | 196.00 | 47.38 | 15.50 | 136.84 | 110.80 | 1.71 | 1.39 |
| Smart | 4.00 |  | 138.50 |  |  |  | 53.92 |  | 1.80 |  |
| Smart | 4.00 | 6.00 | 205.67 | 169.25 | 150.00 | 81.00 | 299.77 | 176.69 | 27.40 | 16.15 |
| Smart | 7.00 |  | 171.08 |  | 86.00 |  | 65.00 |  | 2.17 |  |
| Smart | 5.00 |  | 190.00 |  | 100.00 |  | 280.00 |  | 18.67 |  |
| Smart | 6.00 |  | 141.66 |  | 133.33 |  | 250.00 |  | 2.50 |  |
| Smart | 5.00 |  | 310.00 |  | 75.00 |  | 320.00 |  | 21.33 |  |
| Smart | 4.00 |  | 220.00 |  | 110.00 |  | 140.00 |  | 9.33 |  |
| Control |  |  | 154.83 | 158.50 | 134.17 | 142.83 | 292.51 | 160.59 |  |  |
| Control |  |  | 357.00 | 368.33 | 68.83 | 121.67 | 251.96 | 230.31 |  |  |
| Control |  |  | 185.00 | 189.17 | 149.33 | 153.50 | 321.05 | 180.19 |  |  |
| Control |  |  | 260.33 | 276.83 | 14.67 | 14.17 | 139.80 | 125.67 |  |  |
| Control |  |  | 142.00 | 149.83 | 155.83 | 163.50 | 299.49 | 190.60 |  |  |
| Control |  |  | 232.67 | 242.33 | 134.83 | 135.50 | 301.27 | 296.11 |  |  |
| Control |  |  | 218.17 | 221.33 | 129.00 | 125.83 | 293.73 | 165.81 |  |  |
| Control |  |  | 150.00 | 142.33 | 16.67 | 17.50 | 141.73 | 149.91 |  |  |
| Control |  |  | 233.33 | 245.33 | 83.00 | 76.67 | 118.40 | 125.38 |  |  |
| Control |  |  | 379.83 | 368.17 | 100.33 | 118.67 | 237.48 | 235.35 |  |  |
| Control |  |  | 157.50 | 163.50 | 89.67 | 97.50 | 200.14 | 182.25 |  |  |
| Control |  |  | 143.50 | 144.17 | 96.00 | 97.50 | 219.83 | 98.66 |  |  |
| Control |  |  | 171.00 | 175.00 | 84.33 | 81.67 | 177.77 | 148.02 |  |  |
| Control |  |  | 224.83 | 236.33 | 110.67 | 112.67 | 223.67 | 192.46 |  |  |
| Control |  |  | 126.00 | 133.83 | 133.67 | 147.83 | 284.17 | 197.53 |  |  |
| Control |  |  | 210.17 | 221.50 | 102.67 | 128.00 | 197.79 | 197.87 |  |  |
| Control |  |  | 154.83 | 166.67 | 126.00 | 130.50 | 292.84 | 158.65 |  |  |
| Control |  |  | 228.67 | 245.50 | 136.17 | 147.00 | 237.80 | 231.60 |  |  |
| Control |  |  | 232.67 | 240.50 | 86.00 | 92.00 | 238.63 | 108.31 |  |  |
| Control |  |  | 130.67 | 137.67 | 81.17 | 86.17 | 148.59 | 133.10 |  |  |
| Control |  |  | 140.33 | 143.67 | 124.33 | 136.33 | 192.28 | 185.32 |  |  |
| Control |  |  | 164.67 | 173.00 | 106.00 | 129.50 | 159.70 | 158.40 |  |  |
| Control |  |  | 146.50 | 153.33 |  |  | 72.00 | 46.00 |  |  |
| Control |  |  | 254.50 | 270.33 | 55.50 | 42.33 | 175.58 | 168.22 |  |  |
| Control |  |  | 158.50 | 160.33 | 111.17 | 106.33 | 238.63 | 204.51 |  |  |
| Control |  |  | 331.50 | 352.50 | 77.33 | 81.17 | 307.98 | 275.61 |  |  |
| Control |  |  | 148.17 | 149.00 | 153.83 | 155.50 | 290.02 | 277.30 |  |  |
| Control |  |  | 121.17 | 133.83 | 136.50 | 143.00 | 202.02 | 175.08 |  |  |
| Control |  |  | 226.17 | 243.00 | 101.00 | 111.00 | 187.00 | 202.00 |  |  |
| Control |  |  | 245.00 | 243.83 | 61.17 | 50.00 | 214.36 | 199.11 |  |  |
| Control |  |  | 148.00 | 112.17 | 89.50 | 51.17 | 130.80 | 111.93 |  |  |
| Control |  |  | 223.17 | 237.17 | 102.00 | 104.83 | 215.70 | 200.19 |  |  |
| Control |  |  | 104.17 | 103.33 | 55.33 | 58.83 | 95.05 | 98.39 |  |  |
| Control |  |  | 325.33 | 326.33 | 112.50 | 117.00 | 298.28 | 304.80 |  |  |
| Control |  |  | 79.67 | 86.67 | 73.00 | 76.67 | 86.62 | 91.27 |  |  |
| Control |  |  | 150.50 | 158.17 | 118.33 | 124.33 | 237.65 | 211.65 |  |  |
| Control |  |  | 319.00 | 328.50 | 128.67 | 127.67 | 285.16 | 288.26 |  |  |
| Control |  |  | 129.33 | 136.67 | 58.83 | 61.17 | 79.36 | 65.73 |  |  |
| Control |  |  | 191.50 | 186.67 | 55.67 | 64.33 | 112.15 | 80.22 |  |  |
| Control |  |  | 141.50 | 142.17 | 46.17 | 47.00 | 87.01 | 85.69 |  |  |
| Control |  |  | 130.83 | 142.50 | 70.67 | 74.17 | 137.78 | 138.84 |  |  |
| Control |  |  | 228.00 | 248.17 | 113.00 | 118.00 | 237.21 | 242.08 |  |  |
| Control |  |  | 158.17 | 172.67 | 140.00 | 145.33 | 216.88 | 199.97 |  |  |
| Control |  |  | 243.00 | 248.67 | 146.17 | 157.83 | 272.74 | 257.41 |  |  |
| Control |  |  | 54.33 | 58.17 | 24.33 | 27.00 | 37.68 | 41.00 |  |  |
| Control |  |  | 109.67 | 104.50 | 26.50 | 30.17 | 73.02 | 76.45 |  |  |
| Control |  |  | 152.83 | 154.67 | 87.33 | 96.00 | 139.85 | 143.89 |  |  |
| Control |  |  | 179.50 | 188.67 | 106.67 | 120.83 | 158.77 | 140.27 |  |  |
| Control |  |  | 144.83 | 163.17 | 83.33 | 86.83 | 161.00 | 150.21 |  |  |
| Control |  |  | 115.50 | 131.00 | 48.17 | 65.83 | 84.80 | 86.66 |  |  |
| Control |  |  | 162.67 | 175.67 | 52.67 | 62.00 | 160.72 | 121.21 |  |  |
| Control |  |  | 235.33 | 176.33 | 59.50 | 50.00 | 146.71 | 134.17 |  |  |

Appendix E. Primary and Secondary Building Indicators for Energy Poverty

| **Groups** | **Home Energy Label** | **Home Type** | **Home Size** | **Year of Build** | **Heating Type** | **Cooking Type** | **Window Type** | **Pre test % Income Spent on Energy** | **Post test % Income Spent on Energy** |
| --- | --- | --- | --- | --- | --- | --- | --- | --- | --- |
| Static | D or lower | Terraced House (between and ground) | 94m2 | 1917 | Combi Boiler | Gas | Double Glass | 12.59 | 7.68 |
| Static | D or lower | Terraced House (between and middle) | 92m2 | 1930 | Electric Heating | Electric | Double Glass | 13.61 | 6.81 |
| Static | G | Terraced House (between and top) | 54m2 | 1850 | Central Heating | Gas | Single Glass | 17.11 | 12.77 |
| Static | E | Terraced House (between and ground) | 80m2 | 1918 | District Heating | Gas | Double Glass | 1.85 |  |
| Static | B | Apartment (between and top) | 110m2 | 1977 | Central Heating | Gas | Double Glass | 2.89 | 2.62 |
| Static | E | Terraced House (between and ground) | 105m2 | 1864 | District Heating | Gas | Single Glass | 12.52 | 8.57 |
| Static | G | Apartment (between and middle) | 45m2 | 1892 | Block Heating | Gas | Single and Double Glass | 9.48 | 7.19 |
| Static | E | Apartment (between and middle) | | 1930 | Combi Boiler | Electric | Single and Double Glass | 7.78 |  |
| Static | C | Apartment (between and middle) | 45m2 | 1988 | Combi Boiler | Gas | Double Glass | 3.30 | 1.51 |
| Static | E | Terraced House (between and middle) | 76 m2 | 1914 | Combi Boiler | Gas | Double Glass | 3.06 | 3.27 |
| Static | D | Terraced House (between and ground) | 42m2 | 1925 | Combi Boiler | Gas | Double Glass | 7.47 | 5.02 |
| Static | B | Apartment (between and middle) | 76 m2 | 2002 | Combi Boiler | Electric | Double Glass | 5.75 | 3.67 |
| Static | D or lower | Terraced House (corner and top) | 44m2 | 1994 | Combi Boiler | Electric | Double Glass | 5.17 | 5.17 |
| Static | D or lower | Terraced House (between and middle) | 83m2 | 1901 | Combi Boiler | Gas | Double Glass | 6.62 | 3.77 |
| Static | D or lower | Terraced House (between and middle) | 90m2 | 1888 | Combi Boiler | Gas | Single Glass | 9.79 | 9.18 |
| Static | D | Terraced House (between and middle) | 70m2 | 1911 | Combi Boiler | Electric | Single Glass | 0.16 | 0.15 |
| Static | D or lower | Apartment (between and ground) | 50m2 | 1976 | Central Heating | Gas | Double Glass | 10.00 | 10.00 |
| Static | C | Apartment (between and middle) | 25m2 | 2005 | Combi Boiler | Electric | Double Glass | 7.15 | 7.15 |
| Static | G | Terraced House (between and middle) | 50m2 | 1910 | Combi Boiler | Gas | Double Glass | 2.48 | 1.10 |
| Static | E | Apartment (corner and middle) | 45m2 | 1982 | Combi Boiler | Gas | Double Glass | 10.21 | 5.05 |
| Static | D or lower | Apartment (between and middle) | | 1982 | Combi Boiler | Gas | Double Glass | 0.16 |  |
| Static | D or lower | Terraced House (between and top) | 60m2 | 1913 | Combi Boiler | Gas | Double Glass | 12.23 | 11.87 |
| Static | D or lower | Terraced House (between and ground) | 53m2 | 1905 | Combi Boiler | Gas | Double Glass | 10.57 | 6.81 |
| Static | D | Terraced House (between and middle) | 48m2 | 1910 | Combi Boiler | Gas | Single and Double Glass | 9.63 | 7.74 |
| Static | D | Terraced House (between and middle) | 73m2 | 1925 | Combi Boiler | Gas | Double Glass | 5.79 | 5.13 |
| Static | B | Terraced House (between and top) | 120m2 | 1939 | Combi Boiler | Electric | Double Glass | 4.59 | 3.76 |
| Static | D or lower | Apartment (between and middle) | 68m2 | 1989 | Combi Boiler | Gas | Double Glass | 21.74 | 9.57 |
| Static | D or lower | Terraced House (between and middle) | 40m2 | 1994 | Combi Boiler | Gas | Double Glass | 12.66 |  |
| Static | D or lower | Terraced House (between and top) | 65m2 | 1987 | Combi Boiler | Gas | Double Glass | 11.52 | 7.68 |
| Static | D | Terraced House (corner and ground) | 72m2 | 1990 | Combi Boiler | Electric | Single and Double Glass | 2.55 | 2.88 |
| Static | D or lower | Apartment (between and middle) | 55m2 | 1994 | Combi Boiler | Electric | Double Glass | 6.67 | 6.67 |
| Static | No data | Terraced House (between and ground) | 90m2 | 1930 | Combi Boiler | Gas | Double Glass | 1.26 |  |
| Static | D or lower | Terraced House (between and top) | 120m2 | 1930 | Combi Boiler | Gas | Double Glass | 3.01 |  |
| Static | F | Terraced House (between and corner) | 76m2 | 1963 | Central Heating | Electric | Double Glass | 30.25 |  |
| Static | C | Terraced House (between and middle) | 62m2 | 1900 | Combi Boiler | Gas | Double Glass | 8.58 | 5.33 |
| Static | D | Terraced House (corner and top) | 68m2 | 1901 | Combi Boiler | Gas | Double Glass | 2.41 | 2.35 |
| Static | D | Terraced House (corner and top) | 45m2 | 1907 | Combi Boiler | Gas | Double Glass | 10.70 | 5.63 |
| Static | F | Terraced House (between and middle) | 86m3 | 1929 | Combi Boiler | Gas | Double Glass | 7.50 | 7.80 |
| Static | A | Apartment (between and middle) | 40m2 | 1975 | Block Heating | Electric | HR++ Glass | 33.26 |  |
| Static | A | Apartment (between and middle) | 52m2 | 1975 | Block Heating | Electric | HR++ Glass | 23.13 |  |
| Static | A | Apartment (between and middle) | 72m2 | 1990 | Combi Boiler | Electric | HR++ Glass | 2.00 |  |
| Static | C | Apartment (between and middle) | 35m2 | 1881 | Combi Boiler | Gas | Double Glass | 5.90 |  |
| Static | C | Apartment (between and middle) | 90m2 | 1890 | Combi Boiler | Gas | HR++ Glass | 10.40 |  |
| Static | A | Apartment (between and middle) | 110m2 | 1983 | Combi Boiler | Gas | HR++ Glass | 8.14 |  |
| Static | D | Apartment (between and middle) | 68m2 | 1976 | Central Heating | Gas | Double Glass | 20.00 |  |
| Static | A | Apartment (between and middle) | 70m2 | 1984 | Combi Boiler | Gas | Single and Double Glass | 29.58 |  |
| Static | G | Apartment (between and middle) | 45m2 | 1912 | Combi Boiler | Gas | Single Glass | 2.88 |  |
| Static | C | Terraced House (between and middle) | 67m2 | 1922 | Combi Boiler | Gas | HR++ Glass | 6.86 |  |
| Static | G | Apartment (between and middle) | 96m2 | 1934 | Combi Boiler | Gas | Single Glass | 3.24 |  |
| Static | C | Apartment (between and middle) | 78m2 | 1987 | Combi Boiler | Gas | Double Glass | 3.11 |  |
| Static | C | Terraced House (between and middle) | 70m2 | 1985 | Combi Boiler | Gas | Double Glass | 2.74 |  |
| Static | B | Terraced House (between and middle) | 80m2 | 1985 | Combi Boiler | Gas | HR++ Glass | 10.00 |  |
| Static | A | Terraced House (between and middle) | 76m2 | 1985 | Combi Boiler | Gas | Double Glass | 10.12 |  |
| Static | D or lower | Apartment (between and middle) | 62m2 | 1913 | Combi Boiler | Gas | Single and Double Glass | 10.00 |  |
| Static | D or lower | Apartment (between and middle) | 55m2 | 1899 | Combi Boiler | Gas | Single and Double Glass | 6.00 |  |
| Static | D | Terraced House (corner and middle) | 54m2 | 1939 | Central Heating | Gas | Double Glass | 10.00 |  |
| Static | E | Terraced House (between and middle) | 74m2 | 1939 | Combi Boiler | Gas | Double Glass | 9.50 |  |
| Static | D | Apartment (between and middle) | 38m2 | 1929 | Combi Boiler | Gas | Double Glass | 7.50 |  |
| Static | No data | Apartment (between and middle) | 59m2 | 1924 | Central Heating | Electric | Double Glass | 10.00 |  |
| Static | No data | Apartment (between and middle) | 60m2 | 1995 | Combi Boiler | Gas | Double Glass | 16.67 |  |
| Static | No data | Apartment (between and middle) | 44m2 | 1936 | Combi Boiler | Electric | Double Glass | 34.88 |  |
| Static | B | Apartment (between and middle) | 77m2 | 1994 | Combi Boiler | Gas | Double Glass | 10.46 |  |
| Static | C | Apartment (between and middle) | 50m2 | 1989 | Combi Boiler | Electric | Double Glass | 10.83 |  |
| Static | No data | Apartment (between and middle) | 50m2 | 1929 | Combi Boiler | Gas | Double Glass | 26.54 |  |
| Static | D or lower | Apartment (between and top) | 60m2 | 1990 | Combi Boiler | Electric | Double Glass | 12.67 |  |
| Static | F | Terraced House (between and top) | 60m2 | 1890 | Combi Boiler | Gas | Single Glass | 11.67 |  |
| Static | D or lower | Terraced House (between and middle) | 46m2 | 1914 | Combi Boiler | Gas | Double Glass | 11.00 |  |
| Smart | G | Terraced House (between and middle) | 2x 51m2 | 1936 | Combi Boiler | Electric | Single Glass | 2.01 | 1.81 |
| Smart | D | Terraced House (between and middle) | 51 m2 | 1900 | Combi Boiler | Gas | Double Glass | 3.26 | 2.10 |
| Smart | D | Terraced House (between and ground) | 55m2 | 1983 | Central Heating | Gas | Double Glass | 20.79 | 7.85 |
| Smart | D | Terraced House (between and middle) | No | 1923 | Combi Boiler | Gas | Double Glass | 22.97 | 7.44 |
| Smart | F | Terraced House (corner and middle) | 88m2 | 1899 | Combi Boiler | Gas | Single Glass | 10.94 | 0.06 |
| Smart | D or lower | Terraced House (between and middle) | 62m2 | 1985 | Combi Boiler | Gas | Double Glass | 13.61 | 7.41 |
| Smart | G | Terraced House (between and top) | 80m2 | 1890 | Combi Boiler | Gas | Single and Double Glass | 3.43 | 1.97 |
| Smart | D or lower | Terraced House (between and middle) | 62m2 | 1983 | Combi Boiler | Gas | Double Glass | 21.86 | 7.03 |
| Smart | B | Apartment (between and middle) | 70m2 | 1990 | Combi Boiler | Electric | Double Glass | 3.61 | 2.51 |
| Smart | No data | Apartment (corner and ground) | 64 m2 | 1972 | Combi Boiler | Electric | Double Glass | 8.88 | 5.07 |
| Smart | D or lower | Apartment (between and top) | 50m2 | 1972 | Block Heating | Gas | Double Glass | 18.53 | 7.49 |
| Smart | D | Apartment (corner and between) | 38m2 | 1972 | Combi Boiler | Gas | Double Glass | 13.72 | 6.03 |
| Smart | D | Terraced House (between and middle) | 55m2 | 1933 | Combi Boiler | Gas | Double Glass | 1.68 | 0.74 |
| Smart | D | Terraced House (between and middle) | 60m2 | 1938 | Combi Boiler | Gas | Single Glass | 5.20 | 2.17 |
| Smart | D | Terraced House (between and middle) | 50m2 | 1965 | Combi Boiler | Gas | Double Glass | 28.07 | 13.57 |
| Smart | F | Terraced House (between and middle) | 48m2 | 1922 | Gas Heater | Gas | Double Glass | 14.37 | 5.66 |
| Smart | E | Terraced House (between and corner) | 45m2 | 1939 | Combi Boiler | Gas | Single Glass | 1.16 | 0.75 |
| Smart | C | Apartment (between and middle) | 57m2 | 1990 | Combi Boiler | Electric | Double Glass | 8.94 | 6.32 |
| Smart | D | Terraced House (between and top) | 56m2 | 1987 | Combi Boiler | Gas | Double Glass | 10.43 | 4.06 |
| Smart | F | Terraced House (corner and ground) | 43m2 | 1858 | Combi Boiler | Electric | Double Glass | 4.57 | 2.37 |
| Smart | D | Apartment (between and corner) | 65m2 | 1962 | Combi Boiler | Electric | Double Glass | 41.40 | 18.09 |
| Smart | D | Terraced House (between and middle) | 80m2 | 1997 | Combi Boiler | Gas | Double Glass | 5.57 | 4.94 |
| Smart | F | Terraced House (between and ground) | 58m2 | 1931 | Combi Boiler | Gas | Double Glass | 7.29 |  |
| Smart | F | Terraced House (between and top) | 68m2 | 1934 | Combi Boiler | Electric | Single and Double Glass | 8.26 | 3.35 |
| Smart | F | Terraced House (between and top) | 34m2 | 1905 | Combi Boiler | Electric | Single and Double Glass | 8.01 | 5.31 |
| Smart | G | Terraced House (between and top) | 67m2 | 1933 | Combi Boiler | Gas | Single and Double Glass | 3.02 | 1.71 |
| Smart | F | Terraced House (corner and top) | 72m2 | 1910 | Combi Boiler | Gas | Single Glass | 4.51 | 2.43 |
| Smart | No data | Terraced House (corner and middle) | 80m2 | 1928 | Combi Boiler | Electric | Double Glass | 13.81 | 4.41 |
| Smart | D | Terraced House (between and middle) | 53m2 | 1922 | Combi Boiler | Gas | Double Glass | 8.10 | 8.25 |
| Smart | E | Terraced House (between and middle) | 54m2 | 1934 | Combi Boiler | Gas | Single Glass | 26.83 | 9.99 |
| Smart | C | Terraced House (corner and middle) | 38m2 | 1989 | Combi Boiler | Gas | Double Glass | 3.66 | 1.70 |
| Smart | No | Apartment (between and middle) | 41m2 | 2023 | Electric Heating | Electric | Double Glass | 4.13 | 2.81 |
| Smart | No data | Terraced House (between and ground) | 35m2 | 1905 | Combi Boiler | Gas | HR++ Glass | 11.32 | 3.87 |
| Smart | B | Apartment (between and ground) | 68m2 | 2022 | District Heating | Electric | HR++ Glass | 10.24 | 4.26 |
| Smart | No data | Terraced House (corner and middle) | 80m2 | 1905 | Central Heating | Gas | Single Glass | 9.71 | 4.59 |
| Smart | G | Terraced House (between and ground) | 70m2 | 1933 | Central Heating | Gas | Single and Double Glass | 5.50 | 1.92 |
| Smart | D | Terraced House (between and ground) | 119m2 | 1931 | Combi Boiler | Gas | Single Glass | 1.20 | 0.62 |
| Smart | D or lower | Terraced House (between and ground) | 76m2 | 2006 | Combi Boiler | Gas | Double Glass | 16.79 | 7.41 |
| Smart | C | Terraced House (between and middle) | 75m2 | 1881 | Combi Boiler | Electric | Single and Double Glass | 5.40 |  |
| Smart | G | Terraced House (corner and top) | 76 m2 | 1950 | Combi Boiler | Electric | Double Glass | 8.06 | 3.53 |
| Smart | D | Rijtjeswoning tussen | 69m2 | 1927 | Combi Boiler | Electric | Double Glass | 8.28 | 1.91 |
| Smart | D | Terraced House (between and ground) | 75m2 | 1986 | Combi Boiler | Gas | Single and Double Glass | 1.21 | 0.45 |
| Smart | D or lower | Terraced House (between and ground) | 93m2 | 1986 | Combi Boiler | Gas | Double Glass | 1.71 | 1.39 |
| Smart | D | Apartment (corner and middle) | 100m2 | 1971 | District Heating | Electric | Double Glass | 1.80 |  |
| Smart | D or lower | Terraced House (corner and ground) | 62m2 | 1927 | Combi Boiler | Gas | Single and Double Glass | 27.40 | 16.15 |
| Smart | D | Apartment (between and middle) | 63m2 | 1967 | District Heating | Gas | Double Glass | 2.17 |  |
| Smart | No data | Terraced House (between and top) | 78m2 | 1920 | Combi Boiler | Gas | Single and Double Glass | 18.67 |  |
| Smart | C | Apartment (corner and middle) | 145m2 | 1780 | Combi Boiler | Gas | Double Glass | 2.50 |  |
| Smart | G | Terraced House (between and ground) | 44m2 | 1985 | Combi Boiler | Electric | Double Glass | 21.33 |  |
| Smart | D or lower | Terraced House (between and middle) | 60m2 | 1953 | Combi Boiler | Gas | Double Glass | 9.33 |  |

Appendix F. Primary and Secondary Social Indicators for Energy Poverty

| **Groups** | **Household Income (€)** | **Number of Residents** | **Housing Status** | **Single Income Home** | **Living on Social Allowance (pension or benefits)** | **Ethnic Minority (non-Dutch white)** | **Recent Migrant (less than 5 years in Nl)** | **Older Age (65+ years old)** | **Long term Health issues** | **Single Parent Family** | **% Before** | **% After** |
| --- | --- | --- | --- | --- | --- | --- | --- | --- | --- | --- | --- | --- |
| Static | 1500 | 1 | Social Housing | Yes | Yes |  |  | Yes | Yes |  | 12.59 | 7.68 |
| Static | 3000 | 3 | Owner | Yes |  |  | Yes |  |  |  | 13.61 | 6.81 |
| Static | 1500 | 2 | Renting from Private Landlord | Yes |  |  | Yes |  |  |  | 17.11 | 12.77 |
| Static | 5833 | 1 | Renting from Private Landlord | Yes |  |  |  | Yes |  |  | 1.85 |  |
| Static | 4300 | 2 | Owner |  | Yes |  |  | Yes |  |  | 2.89 | 2.62 |
| Static | 2705 | 1 | Social Housing | Yes |  |  |  | Yes | Yes |  | 12.52 | 8.57 |
| Static | 2000 | 2 | Social Housing | Yes | Yes |  |  |  |  | Yes | 9.48 | 7.19 |
| Static | 3600 | 1 | Owner |  |  |  | Yes |  |  |  | 7.78 |  |
| Static | 4700 | 1 | Owner | Yes |  |  | Yes |  |  |  | 3.30 | 1.51 |
| Static | 4600 | 1 | Owner | Yes |  | Yes | Yes |  |  |  | 3.06 | 3.27 |
| Static | 2800 | 1 | Owner | Yes |  | Yes |  |  |  |  | 7.47 | 5.02 |
| Static | 2500 | 2 | Owner |  |  | Yes | Yes |  |  |  | 5.75 | 3.67 |
| Static | 1800 | 1 | Social Housing | Yes | Yes |  | Yes |  |  |  | 5.17 | 5.17 |
| Static | 6000 | 2 | Owner | Yes |  | Yes |  |  |  |  | 6.62 | 3.77 |
| Static | 3300 | 3 | Renting from Private Landlord | | Yes |  |  | Yes |  |  | 9.79 | 9.18 |
| Static | 100000 | 1 | Owner | Yes |  |  | Yes |  |  |  | 0.16 | 0.15 |
| Static | 1500 | 2 | Social Housing | Yes | Yes | Yes |  |  |  | Yes | 10.00 | 10.00 |
| Static | 2015 | 1 | Social Housing | Yes |  | Yes | Yes |  |  |  | 7.15 | 7.15 |
| Static | 5833 | 1 | Owner | Yes |  |  | Yes |  |  | Yes | 2.48 | 1.10 |
| Static | 1700 | 1 | Social Housing | Yes | Yes | Yes |  |  | Yes |  | 10.21 | 5.05 |
| Static | 1035 | 2 | Social Housing | Yes | Yes | Yes |  |  |  | Yes | 0.16 |  |
| Static | 1833 | 1 | Renting from Private Landlord | Yes | Yes |  |  |  | Yes |  | 12.23 | 11.87 |
| Static | 1100 | 1 | Renting from Private Landlord | Yes | Yes |  |  | Yes |  |  | 10.57 | 6.81 |
| Static | 1300 | 2 | Social Housing | Yes | Yes | Yes | Yes |  |  | Yes | 9.63 | 7.74 |
| Static | 2500 | 1 | Owner | Yes | Yes |  |  |  | Yes |  | 5.79 | 5.13 |
| Static | 5000 | 3 | Social Housing | |  |  |  |  |  |  | 4.59 | 3.76 |
| Static | 1300 | 1 | Social Housing | Yes | Yes | Yes |  | Yes | Yes |  | 21.74 | 9.57 |
| Static | 1250 | 1 | Social Housing | Yes | Yes |  |  | Yes |  |  | 12.66 |  |
| Static | 1180 | 1 | Social Housing | Yes | Yes |  |  | Yes | Yes |  | 11.52 | 7.68 |
| Static | 4500 | 2 | Renting from Private Landlord | | |  |  |  |  |  | 2.55 | 2.88 |
| Static | 1500 | 1 | Social Housing | |  |  |  | Yes | Yes |  | 6.67 | 6.67 |
| Static | 20000 | 2 | Owner |  |  |  |  |  |  |  | 1.26 |  |
| Static | 10000 | 3 | Renting from Private Landlord | | |  |  |  |  |  | 3.01 |  |
| Static | 1100 | 2 | Owner | Yes | Yes |  |  |  |  |  | 30.25 |  |
| Static | 1500 | 2 | Social Housing | Yes |  |  |  |  | Yes | Yes | 8.58 | 5.33 |
| Static | 2500 | 2 | Owner |  |  |  | Yes |  |  |  | 2.41 | 2.35 |
| Static | 1200 | 1 | Renting from Private Landlord | Yes | Yes |  |  |  | Yes |  | 10.70 | 5.63 |
| Static | 2400 | 2 | Owner |  |  |  | Yes |  |  |  | 7.50 | 7.80 |
| Static | 935 | 2 | Social Housing | Yes |  |  |  | Yes |  |  | 33.26 |  |
| Static | 1600 | 1 | Social Housing | Yes |  | Yes |  | Yes |  |  | 23.13 |  |
| Static | 5000 | 2 | Owner |  |  | Yes |  |  |  |  | 2.00 |  |
| Static | 1101 | 1 | Social Housing | Yes |  | Yes |  |  |  |  | 5.90 |  |
| Static | 1500 | 2 | Renting from Private Landlord | Yes |  |  |  |  |  |  | 10.40 |  |
| Static | 5034 | 4 | Owner |  |  |  |  |  |  |  | 8.14 |  |
| Static | 1500 | 3 | Social Housing | Yes | Yes | Yes |  |  |  |  | 20.00 |  |
| Static | 960 | 2 | Social Housing | Yes | Yes | Yes |  | Yes |  |  | 29.58 |  |
| Static | 2083 | 1 | Renting from Private Landlord | | |  |  |  |  |  | 2.88 |  |
| Static | 1632 | 1 | Social Housing | Yes | Yes |  |  |  |  |  | 6.86 |  |
| Static | 8000 | 4 | Renting from Private Landlord | | |  |  |  |  |  | 3.24 |  |
| Static | 4500 | 3 | Renting from Private Landlord | | | Yes |  |  |  |  | 3.11 |  |
| Static | 2150 | 1 | Social Housing | | Yes |  |  | Yes |  |  | 2.74 |  |
| Static | 3000 | 5 | Owner |  |  |  |  |  |  |  | 10.00 |  |
| Static | 1700 | 1 | Social Housing | Yes | Yes | Yes |  |  |  |  | 10.12 |  |
| Static | 1200 | 1 | Social Housing | Yes |  |  |  |  |  |  | 10.00 |  |
| Static | 2500 | 1 | Owner |  |  |  |  |  |  |  | 6.00 |  |
| Static | 1500 | 1 | Social Housing | Yes | Yes |  |  | Yes |  |  | 10.00 |  |
| Static | 2000 | 1 | Social Housing | |  |  |  |  |  |  | 9.50 |  |
| Static | 4000 | 4 | Renting from Private Landlord | Yes |  |  |  |  |  |  | 7.50 |  |
| Static | 1500 | 1 | Owner | Yes | Yes |  |  | Yes |  |  | 10.00 |  |
| Static | 1200 | 1 | Social Housing | Yes | Yes | Yes |  | Yes |  |  | 16.67 |  |
| Static | 860 | 1 | Social Housing | Yes | Yes |  |  |  |  |  | 34.88 |  |
| Static | 1434 | 1 | Social Housing | Yes | Yes | Yes |  |  |  |  | 10.46 |  |
| Static | 3000 | 1 | Owner | Yes |  |  |  |  |  |  | 10.83 |  |
| Static | 1036 | 1 | Social Housing | Yes | Yes |  |  |  |  |  | 26.54 |  |
| Static | 1500 | 1 | Social Housing | Yes |  |  |  |  |  |  | 12.67 |  |
| Static | 1500 | 1 | Social Housing | Yes | Yes |  |  | Yes |  |  | 11.67 |  |
| Static | 1500 | 1 | Social Housing | Yes | Yes |  |  | Yes |  |  | 11.00 |  |
| Smart | 5000 | 1 | Owner | Yes | Yes |  |  | Yes |  |  | 2.01 | 1.81 |
| Smart | 3000 | 2 | Social Housing | |  |  | Yes |  |  |  | 3.26 | 2.10 |
| Smart | 1320 | 5 | Owner | Yes | Yes | Yes |  | Yes | Yes | Yes | 20.79 | 7.85 |
| Smart | 1150 | 1 | Social Housing | Yes | Yes | Yes |  |  | Yes |  | 22.97 | 7.44 |
| Smart | 1500 | 2 | Renting from Private Landlord | Yes |  |  | Yes |  |  |  | 10.94 | 0.06 |
| Smart | 1900 | 2 | Social Housing | | Yes | Yes |  | Yes | Yes |  | 13.61 | 7.41 |
| Smart | 2500 | 1 | Owner | Yes |  |  |  |  |  |  | 3.43 | 1.97 |
| Smart | 1383 | 1 | Social Housing | Yes | Yes |  |  |  |  |  | 21.86 | 7.03 |
| Smart | 6000 | 2 | Owner |  |  |  |  |  |  |  | 3.61 | 2.51 |
| Smart | 1000 | 1 | Social Housing | Yes | Yes | Yes |  |  | Yes |  | 8.88 | 5.07 |
| Smart | 1050 | 1 | Social Housing | Yes | Yes | Yes |  |  | Yes |  | 18.53 | 7.49 |
| Smart | 1100 | 1 | Social Housing | Yes | Yes | Yes |  | Yes |  |  | 13.72 | 6.03 |
| Smart | 5000 | 1 | Owner | Yes |  |  | Yes |  |  |  | 1.68 | 0.74 |
| Smart | 2500 | 1 | Renting from Private Landlord | Yes |  |  |  | Yes |  |  | 5.20 | 2.17 |
| Smart | 1200 | 1 | Social Housing | Yes | Yes | Yes |  | Yes |  | Yes | 28.07 | 13.57 |
| Smart | 1400 | 1 | Renting from Private Landlord | Yes | Yes |  |  | Yes | Yes |  | 14.37 | 5.66 |
| Smart | 11000 | 2 | Owner |  |  | Yes |  |  |  |  | 1.16 | 0.75 |
| Smart | 1300 | 5 | Social Housing | Yes |  | Yes |  |  |  |  | 8.94 | 6.32 |
| Smart | 2500 | 1 | Owner | Yes |  |  |  |  |  |  | 10.43 | 4.06 |
| Smart | 3700 | 1 | Social Housing | Yes | Yes |  |  |  | Yes |  | 4.57 | 2.37 |
| Smart | 949 | 6 | Social Housing | Yes | Yes |  |  |  | Yes | Yes | 41.40 | 18.09 |
| Smart | 1300 | 1 | Social Housing | Yes | Yes | Yes |  | Yes |  |  | 5.57 | 4.94 |
| Smart | 4000 | 2 | Owner | Yes |  |  |  |  |  |  | 7.29 |  |
| Smart | 2800 | 2 | Renting from Private Landlord | | Yes |  |  |  |  |  | 8.26 | 3.35 |
| Smart | 900 | 1 | Social Housing | Yes | Yes |  |  |  |  |  | 8.01 | 5.31 |
| Smart | 4000 | 1 | Owner | Yes |  |  | Yes |  |  |  | 3.02 | 1.71 |
| Smart | 5000 | 4 | Renting from Private Landlord | | |  |  |  |  |  | 4.51 | 2.43 |
| Smart | 1500 | 2 | Renting from Private Landlord | Yes |  |  |  |  |  |  | 13.81 | 4.41 |
| Smart | 2096 | 3 | Social Housing | Yes | Yes |  | Yes |  | Yes | Yes | 8.10 | 8.25 |
| Smart | 2500 | 2 | Owner | Yes |  |  |  |  |  |  | 26.83 | 9.99 |
| Smart | 3000 | 1 | Owner |  |  | Yes | Yes |  |  |  | 3.66 | 1.70 |
| Smart | 3300 | 1 | Renting from Private Landlord | Yes |  |  | Yes |  |  |  | 4.13 | 2.81 |
| Smart | 1100 | 2 | Social Housing | | Yes |  |  |  | Yes |  | 11.32 | 3.87 |
| Smart | 4800 | 1 | Renting from Private Landlord | | | Yes | Yes |  |  |  | 10.24 | 4.26 |
| Smart | 3600 | 2 | Renting from Private Landlord | Yes |  |  |  |  |  |  | 9.71 | 4.59 |
| Smart | 6000 | 3 | Owner |  |  |  | Yes |  |  |  | 5.50 | 1.92 |
| Smart | 10000 | 4 | Renting from Private Landlord | | | Yes | Yes |  |  |  | 1.20 | 0.62 |
| Smart | 1200 | 1 | Social Housing | Yes | Yes | Yes |  |  | Yes | Yes | 16.79 | 7.41 |
| Smart | 3000 | 4 | Owner |  |  |  | Yes |  |  |  | 5.40 |  |
| Smart | 4000 | 2 | Owner |  |  |  | Yes |  |  |  | 8.06 | 3.53 |
| Smart | 1400 | 2 | Owner | Yes |  |  | Yes |  |  |  | 8.28 | 1.91 |
| Smart | 10000 | 1 | Owner | Yes |  |  |  |  |  |  | 1.21 | 0.45 |
| Smart | 8000 | 2 | Owner | Yes |  |  |  |  |  |  | 1.71 | 1.39 |
| Smart | 3000 | 2 | Renting from Private Landlord | | |  | Yes |  |  |  | 1.80 |  |
| Smart | 1094 | 1 | Social Housing | Yes | Yes |  |  | Yes | Yes |  | 27.40 | 16.15 |
| Smart | 3000 | 1 | Owner |  |  |  |  |  |  |  | 2.17 |  |
| Smart | 1500 | 1 | Social Housing | Yes | Yes | Yes |  | Yes | Yes |  | 18.67 |  |
| Smart | 10000 | 1 | Owner | Yes |  | Yes |  |  |  | Yes | 2.50 |  |
| Smart | 1500 | 1 | Social Housing | Yes | Yes |  |  |  | Yes |  | 21.33 |  |
| Smart | 1500 | 1 | Social Housing | Yes | Yes | Yes |  |  | Yes |  | 9.33 |  |
