# Supplementary material for: Assessing the impact of energy coaching with smart technology interventions to alleviate energy poverty
Source: Sci Rep. 2025 Jan 13;15:969. doi: 10.1038/s41598-024-80773-9 (PMC11730312; doi:10.1038/s41598-024-80773-9)
Supplement: Supplementary file 3 — Supplementary Material 3 [file 41598_2024_80773_MOESM3_ESM.docx]

Appendices

**Appendix A. Energy Coach in Home Assessment Protocol (including questions and advice)**

One day prior to the visit:

Email the home, ask to make sure they have monthly energy bills ready for the visit.

Once at the home:

-Introduce yourself, the organisation and the research project.

“Usually the organization only provides a 1-time visit, however this research looks at the impact of the energy coaching over time.

-Introduce the research consent form, if they wish to participate (ask to record, take pictures of windows, the layout of the room and jot down some notes during the visit too).

“We will go through the home and try to answer any and all questions you have”

-Inform the home that all advice and products are free. Take the products out on the table before beginning the survey questions.

“I have some of these products for you, which will help improve the efficiency of the home. They are small scale measures that create larger efficiency gains over time. I can install them myself during this visit. If you have any reservations and want to do some larger scale renovations and retrofits (i.e. changing out the windows or installing heat pumps) I can refer you to our legal team with information, or other organizations that could help. I can install a smart energy display in the home also. The smart energy display gives real time feedback on your consumption, income for energy and the efficiency of your home”.

- Once complete with questions, advice and installations, inform the homes of next steps.

“You will receive a report with tailored energy advice and information for yourself. You are free to use this information how you like. I am able to revisit your home again in 6 months to see how you’re getting on, if you need any further help and whether or not the report, products or smart display have changed energy-use in your home. I will leave my contact information too if you have any questions in the meantime”.

**Standardised Questions and Advice**

**1.General Information**

1A. Date of Home

B. Name of energy coach(es)

C. Type of conversation Home visit or video call

D. Where do you know the Energy Coach Project from? *not required

- -!Woon
- -Municipality of Amsterdam (website)
- -Municipality of Amsterdam - city pass
- -The energy coach
- -My landlord
- -Our homeowners' association
- -Advertising (flyer, poster, social media, newspaper)
- -Through acquaintances (neighbors, friends, family)
- -Via a service provider (Neighborhood Team, administrator, other)
- -Other

E. Which services did you use last year? *multiple answers possible

- -door-to-door advice from Climate Route
- -door-to-door advice from Energiebox
- -door-to-door advice from the Energy Savings Service (from Profit from your Home)
- the FIX Brigade
- -energy advice from the Neighborhood Team
- -yes, but I don't know (exactly) who
- -no

**2. Home characteristics and household**

1. What type of home do you live in?

- -Apartment
- -Terraced house between
- -Terraced house corner / semi-detached house
- -Detached house

2. A. Year of construction of home

2. B. Are the exterior walls better insulated after 1988?

- -Yes
- -No
- -I do not know

3. How many people does your household consist of?

4. A. Are you a renter or owner-occupier?

- -Tenant

4. B. Who do you rent from?

- -Housing association

4.C Select your housing co-operation

- -Private landlord

4.C Name your landlord

- -Owner-occupier

4.B Is your home part of an owners association?

- -Yes

4. C. Name of Owners Association

4. D. How large is your Home Owners Association?

- - - - 1-10 homes
      - 11-25 homes
      - 26-50 homes
      - More than 50 homes

4. E. Does the Owners' Association consist of more than half of tenants or buyers?

- - - - Tenants
      - Owner-occupiers
      - I do not know
- -No
- -Don’t know

**3. Heating and Energy Data**

1A. What kind of space heating do you have?

- -Gas Heater

1B. How do you cook?

- - - On gas
    - Electric (induction or ceramic)
- -VR Boiler (Mother fireplace with radiators or old central heating)

TIP! Use the free services of the !WOON Foundation to obtain a better heating system! Your living comfort increases and you save considerably on your energy bill. For tenant: * If you are willing to pay a rent increase, a landlord is obliged to cooperate with the proposal *Would you like support in arranging a better heating installation? For owner-occupier in a WE *Do you live in a homeowners' association? Then coordination with the other owners is necessary. For owner-occupiers without a homeowners' association: * You can install a better heating system yourself. Do you want more information?

1. B. Would you like to receive an email with links to the home improvement page?

- Yes
- No

2. Do you have a maintenance contract for your boiler or do you have it regularly checked depending on the instructions of the manufacturer of your boiler?

- Yes
- No

3. How do you cook?

- - - On gas
    - Electric (induction or ceramic)

TIP! This app calculates what you can save. The app calculates the rates below the price ceiling. Please note: The possible savings resulting from this questionnaire are an indication. You may be able to save more or less, depending on your situation. For example, if you have a contract with lower rates than those mentioned above, you simply pay your lower rates. Do you have a contract with higher rates? Then you pay the rates mentioned above until the ceiling is reached and only then your contractual rate. The temporary price ceiling only applies in 2023. If you have more questions about how it works for block heating, joint connections or the distribution over a year, please visit the government website: https://www.rijksoverheid.nl/onderwerpen /koopkracht/spelen-kabinet-met Prijsplafond-voor-gas-en-elektriciteit

- -HR Combi-boiler

1b. Do you have a maintenance contract for your boiler or do you have it regularly checked depending on the instructions of the manufacturer of your boiler?

- Yes
- No

2. Do you have underfloor heating in the living room?

- Yes
- No

3. How do you cook?

- - - On gas
    - Electric (e.g. induction or ceramic)
- -District Heating

2. Do you have underfloor heating in the living room?

- Yes
- No

3. How do you cook?

- - - On gas
    - Electric (e.g. induction or ceramic)
- -Block Heating

2. Do you have underfloor heating in the living room?

- Yes
- No

3. How do you cook?

- - - On gas
    - Electric (e.g. induction or ceramic)
- -Heating with Electric Supply

TIP! The actual savings for the electrical supply is difficult to determine, because your home is better insulated than the average home. Your consumption and therefore the savings will very possibly be lower than we can calculate. The calculations are based on a complete heat pump. If you have a hybrid heat pump, approximately one third of your consumption consists of gas. This consumption does emit extra CO2, but this is considerably less than with a full gas supply.

2. Do you have underfloor heating in the living room?

- Yes
- No

3. How do you cook?

- - - On gas
    - Electric (e.g. induction or ceramic)

TIP! This app calculates what you can save. The app calculates the rates below the price ceiling. Please note: The possible savings resulting from this questionnaire are an indication. You may be able to save more or less, depending on your situation. For example, if you have a contract with lower rates than those mentioned above, you will simply pay your lower rates. Do you have a contract with higher rates? Then you pay the rates mentioned above until the ceiling is reached and only then your contractual rate. The temporary price ceiling only applies in 2023. If you have more questions about how it works for block heating, joint connections or the distribution over a year, please visit the government website: https://www.rijksoverheid.nl/onderwerpen /koopkracht/spelen-kabinet-met -prijsplafond-voor-gas-en-elektriciteit

1. Do you have solar panels for your own supply?

- Yes, for all my electricity consumption
- Yes, for part of my electricity consumption
- No

2. Do you purchase green energy through your supplier?

- Yes

-Yes and Yes.TIP! How great that you generate your electricity with your own solar panels and supplement the rest with green energy! This way you contribute to reducing CO2 emissions. For all calculations in app, the kg of CO2 savings is set to 0.

- No

-Yes and No.TIP! How great that you generate your electricity with your own solar panels! This way you contribute to reducing CO2 emissions. When calculating in this app for electrical equipment, the kg of CO2 savings is set to 0. You purchase gray energy through your supplier. This means that you emit 100% CO2 for heating.

-Partly Yes and No. TIP! How great that you generate part of your electricity with your own solar panels! This way you contribute to reducing CO2 emissions. We assume that you have approximately 80 percent of your generates its own electricity supply, so only 20 percent of the total CO2 emissions. In the calculations in this app for electrical equipment, the kg of CO2 savings is set at 20 percent. You purchase gray energy through your supplier. This means that you emit 100% CO2 for heating.

- I do not know

3. How much energy do you use in a year?

- It was not possible to request the annual consumption.

Consumption:

- Enter total kWh
- Enter total m3

TIP! The average electricity use for a household of X people is X kWh per year. Your usage is therefore X than average. TIP! The average gas consumption for a X house is X m3 per year. Your consumption is therefore X than average.

**4. Heating & Cooling**

1. How many degrees do you set the heating to when it is cold? (Enter a number between 10 and 30 degrees)

TIP! You will save 120.51 euros per year per degree that you reduce your heating! In total, if you heat at 21 degrees, you save 482.04 euros, 430.39 m³ and 766.10 kg of CO2.

2. Do you only heat the room where you are in them and close the interior doors?

- Yes always
- Sometimes

TIP! Only heat the rooms you use and close the interior doors. This saves you 180.76 euros, 161.40 m³ and 287.29 kg of CO2 per year!

- No never

Tip! Only heat the rooms you use and close the interior doors. This saves you 361.53 euros, 322.79 m³ and 574.57 kg of CO2 per year!

3. When you go to bed in the evening, do you turn the heating down or even turn it off completely?

- Yes always
- Sometimes
- No never

4. A. Do you provide additional heating?

- Yes, with a radiator heater

TIP! Pay attention to the power of electric heaters. Radiant heaters, blower heaters, oil-filled radiators and electric radiators often use 1,500W to 2,500W. If you leave these hours on, it will actually cost you more than with your normal heating. If you want to heat locally (for example the workplace at home) or briefly (for example the bathroom), it is economical to heat with infrared.

- Yes, with infrared panels

TIP! How good that you have chosen infrared panels. This uses much less power than with a radiant heater.

- Yes, with underfloor heating in the bathroom
- No

1. Do you have radiators in the home?

- Yes

2. A. Have you applied radiator foil to radiators that are placed on outside walls?

- Yes
- No not yet

2. B. Approximately how many meters of radiator foil do you need? (only for radiators on the outside walls, the foil is 50 cm high) (Enter a number between 1 and 20 meters, make an estimate, do not measure!)

- No, not possible with my type of radiators

Tip! You may still be able to save with foil other than the one the energy coach carries. Get information from a hardware store or specialist.

3. Is there furniture or curtains hanging in front of the radiators?

- Yes

Tip! Do not place furniture or hang curtains in front of radiators. The heat is then absorbed into the furniture or curtains and the room is not heated properly.

- No

4. A. Do your radiators sometimes make ticking noises?

- Yes

TIP! Bleed the radiators. If there is air in the radiators, the heating will work less well. You can also feel this because parts of the radiator do not heat up properly. There are many good instructional videos on the internet on how to do this for your heating type. This saves you 30.00 euros, 38.00 m³ and 67.00 kg of CO2 per year! Check the pressure of your boiler after bleeding it. This must be between 1 and 2 bar.

4B. Do you want a radiator air vent?

- - Yes
  - No
- No

1. Do you cool in the summer?

- No
- Yes, with fan(s)
- Yes, with (mobile) air conditioning
- Yes, but I have other cooling (for example via the Overheating Source, as is sometimes possible with a heat pump or WKO)

TIP! The most important thing is to keep the heat out in the summer. When the sun shines on your windows, it can become quite warm in the house. You can place blinds or a parasol in front of your window or close the curtains. Keep doors and windows closed when it is warm. When it cools down at night, you can open the windows together to cool down the house. A fan only moves the air. To cool the air, a bottle of frozen water can be placed in front of it. Air conditioning costs a lot of electricity and can only be done in combination with solar panels. Cooling with underfloor heating is the most energy efficient, but there are few homes with these techniques.

**5. Ventilation & draft**

1. Do you air your home for at least 10 minutes every day? (When airing, you open windows and doors against each other. When ventilating, fresh air is continuously supplied and extracted.)

- Yes always
- Sometimes
- No never

Sometimes or No. TIP! Air your home for at least 10 minutes every day. This saves you heating costs and reduces the chance of mold formation. That is good for your health. Remember to also ventilate well. The better insulated the home is, the more important it is. After all, there are no more seams and cracks through which fresh air comes in.

2. Which type of ventilation do you have?

- System A Natural ventilation (all) ventilation is done by opening windows, grilles and possibly. cracks)
- System C Mechanical ventilation (the air is mechanically extracted in the bathroom, toilet & kitchen, the natural input goes through grilles in the facade/near the

TIP! You can recognize mechanical ventilation by the exhaust valves. You have a button to set this: 1 = not at home, 2 = at home, 3 = while cooking or showering. Never block grilles!

- System D Balance ventilation (with heat recovery) (all air is supplied and extracted mechanically)

TIP! You not only have drain valves in your home, but also supply valves in the living room and bedrooms. These supply valves supply fresh air. With balanced ventilation, this fresh outside air is first heated with the warm extracted air. That is why this system is energy efficient; less heating is required. You have a button to set the ventilation: 1 = not at home, 2 = at home, 3 = while cooking or showering. With balanced ventilation it is better not to ventilate (the heat recovery will then be lost) and never block the grilles!

1. Do you have draft excluders on windows and doors?

- Yes, and still in good condition
- Yes, but needs to be replaced
- No not necessary
- No not yet

2. Do you have draft sills under the exterior doors?

- Yes, and still in good condition
- Yes, but needs to be replaced
- No not yet
- No not necessary

TIP! Do not use draft sills under the doors between the rooms, they often have a little space underneath, which is good for air circulation. Only use draft sills on doors that close off a cold space, such as the balcony, garden, stairwell or an unheated space, such as a utility room or storage space.

3. Do you have a letterbox brush?

- Yes, and still in good condition
- Yes, but needs to be replaced
- No not yet
- No not necessary

TIP! Install draft excluders, draft sills and letterbox brushes where applicable. You save 24.86 euros per year per draft measure! In total you save 49.71 euros, 44.38 m³ and 79.00 kg of CO2 per year!

1. A. Do you have curtains?

- Yes, thick curtains

1. B. Are these closed at night?

- Yes
- No
- Sometimes

TIP! Always close window coverings at night. This provides extra insulation. This means you have to burn less and save money!

- Yes, but no thick curtains, at least not everywhere
- No, no window coverings

TIP! Hang insulating window coverings in the home, for example thick curtains or blackout (roller) curtains. These work well as insulation. This means you have to burn less and save money! You can achieve this much less with other window coverings, such as net curtains, blinds or slats. No, no window coverings

2. What type of glass do you have at home?

- Single glass
- Single and double glazing

TIP! New double HR++ glass insulates 5x better than single glass. Your living comfort increases and you save considerably on your energy bill. For tenant: * If you are willing to pay a rent increase, a landlord is obliged to cooperate with the proposal * In an average Amsterdam rental home, double glazing can be achieved for a rent increase of approximately 20 euros per month * Get started with the step-by-step plan for better home insulation via www.wooninfo.nl/overzicht For owner-occupier in a homeowners' association: *Do you live in an homeowners' association? Then coordination with the other owners is necessary. Double glazing in an apartment is subject to a service charge increase of +/-30 euros per month that expires after 10 years. You can also pay the amount in one go to invest. Take a look at www.wooninfo.nl/VvE information For owner-occupiers without a homeowners' association: * You can install double glazing yourself. Do you want more information? Go to the website [www.regionaalenergieloket.nl/amsterdam](http://www.regionaalenergieloket.nl/amsterdam)

3. Would you like to receive an email with links to the home insulation page?

- - - Yes, I want more information
    - No, I don't want double glazing
- Double glazing
- HR++ glass
- Triple glazing

**6. Hot water use**

1. A. What type of hot water heating do you have? (If you have multiple facilities, choose the one that you use for showering)

- Kitchen geyser

1B. Is the kitchen geyser open or closed?

- - Open

TIP! Open kitchen water heaters may no longer be sold for safety reasons. If you rent your water heater, discuss this with the landlord. If the water heater is yours, consider replacing it.

- - Closed
- Electric boiler
- Other heating with electric supply
- HR Combi Boiler
- District Heating (Heat Exchanger)
- Block Heating

1. A. What kind of shower head is in the bathroom?

- Standard shower head
- Water saving shower head (saving shower)
- Comfort shower head (rain shower)

1. B. Is there a bathtub?

- Yes, and it is also used
- Yes, but it's not being used
- No

2. A. How long do you spend in the shower? (Family members Shower per week Average duration) Enter per family member

TIP! Savings with water saving shower head and/or taking a 5-minute shower Name If economy shower.

TIP!If you implement both measures, you will save a total of X euros, X Gj, X kg CO2 and X liters of water per year! When calculating the savings if you shower for a maximum of 5 minutes, it is assumed that you are using or will be using an economy shower.

2. B. Would you like a timer that allows you to time your shower to 5 minutes?

- Yes
- No

2. C. Are you interested in a water limiter for the shower head or a water-saving shower head?

- Yes, a water-saving shower head (saving shower)
- Yes, a water restrictor
- No

3. Are you interested in water savers for the kitchen and toilet tap?

- Yes
- No
- No not necessary

**7. Electrical appliances**

1. Do you turn off appliances when you are not using them?

- Yes
- No
- Sometimes

2. Do you leave devices on standby when you are not using them?

- Yes
- No
- Sometimes

3. Do you leave device chargers plugged in? Chargers that get warm still use energy when you're not using them. Fortunately, the latest chargers for phones and tablets usually no longer do that.

- Yes
- No
- Sometimes

4. Are you using a power strip with an on/off button to turn off multiple devices at on we ce?

- Yes
- No

TIP! Devices with a standby mode such as televisions, stereo systems and computer printers also use power in standby mode. Older phone chargers or other devices that you are not charging can also use power. This can amount to an average of 450 kWh per year. Limit standby consumption in your home by connecting these devices to a power strip with a switch.

5. Do you pay attention to the energy label when purchasing new electrical appliances?

- Yes
- No

TIP! It is wise to look carefully at the energy label when purchasing new electrical appliances. Devices with a better label are often more expensive to purchase, but consume much less energy. For example: A 25-year-old fridge-freezer uses about 500 kWh per year, and the most economical 73 kWh. That saves almost €100 per year!

1. A. Do you have a washing machine?

- Yes
- No

1. B. Do you fill the washing machine full or half full?

- Often half full
- Sometimes half full

TIP! Always fill your washing machine. This means you have to wash less often and save more money.

- Always full

1. C. At what temperature do you usually wash?

- 15 (not possible with every detergent, but it is 75% more economical than 60)
- 30
- 40
- 60
- 90

TIP! Washing machine manufacturers recommend washing laundry at 60 or 90 degrees once a month to extend the life of the washing machine. In addition, wash your laundry at 30 degrees as much as possible for a better environment.

2. A. Do you have a clothes dryer?

- Yes
- No

2. B. What kind of dryer do you have?

- old label A++ or A+++ / new label B
- old label A or A+ / new label C/D
- no label/older than 10 years

TIP!Choose an automatic program, which is more economical than setting a time. If possible, use a low temperature or eco program. This takes longer, but is more economical and better for your laundry.

2. C. How many drying cycles do you run per week?

TIP! If you halve the number of drying cycles to x, you will save euros, kWh and kg of CO2 per year. It is even better to get rid of the dryer completely and from now on dry your laundry on a clothes line or rack. This now saves you euros, now kWh and now kg of CO2 per year! Air your home daily when you dry the laundry indoors. Otherwise your home will become damp and mold may develop. That is bad for your health.

3. Do you have a dishwasher?

- Yes
- No

1. Does the resident use LED lamps for all lamps?

- Yes
- No

2. Which lamps can the resident save and which ones yield savings?

Wattage Type:

Total:

Wattage

Light bulb type: Light bulb, Energy Saving Lamp (LED), Halogen

Savings: 0.00

TIP! In total you save x euros, x kWh and x kg of CO2! We recommend that you immediately replace your lamps with LED lamps. This allows you to start saving immediately.

**8. Your savings overview**

TIP! You may have been offered services by the !WOON Foundation while completing the questionnaire. These are indicated below with (D). If you fill in your contact details at the end of the questionnaire, an employee of the !WOON Foundation will contact you. Otherwise, you must contact us yourself to use the services.

3 main areas;

This saves you (€)

Energy use (M3 gas/KwH)

CO2 reduction (Kg)

**Heating**

-Set the heating thermostat to a maximum of 21 degrees. This saves you €X Xm3 X kg

-Only heat the rooms that you use and close the interior doors.Smart! You already do this.

-Set the heating to 15 degrees half an hour before going to sleep, or turn it off completely. Smart! You already do this.

-Do not use electric heaters for additional heating. Smart! You already do this.

-Air your home for at least 10 minutes every day. Outside air is less humid than indoor air and can therefore be heated more quickly. This saves you energy and any chance of mold formation is smaller. That is good for your health.

-Install draft excluders on windows and doors with gaps. You save €X,Xm3, X kg. Smart! You have already installed draft excluders or you do not need them.

-Hang insulating window coverings in the home. Smart! You have already done this.

-(D) Be informed about the options for obtaining double glazing. You did not choose this. If you still want this later, you can always contact the Foundation !Woon.

**Radiators**

-Attach radiator foil behind the radiators. You may still be able to save with foil other than the one the energy coach carries. Get information from a hardware store or specialist.

-Do not place furniture close to radiators. The heat then settles in the furniture and the room is not heated properly.

-Bleed the air every six months radiators. This saves you €X, Xm3, xkg

**Shower**

-Showering behavior, Name, Number, Average time: Resident 1, Resident 2

-Use a water restrictor or a new water saving, shower head. This saves you €X, XGj, Xkg. Moreover, this saves you X liters of water!

-Shower for a maximum of 5 minutes per shower. This saves you €X, XGj, Xkg. Moreover, this saves you X liters of water!

-Place a water restrictor on the kitchen and toilet tap. Smart! You have already done this.

**Devices**

-Turn off appliances when you are not using them. This can save you more money than you think.

-Don't leave any devices with a standby mode on if you do not use them in standby mode. Devices such as televisions, computers and stereos also use power in standby mode.

-Unplug chargers when you are not charging the devices. Phone chargers or other devices that you are not charging can also use a lot of power.

-Pay attention to the energy label when purchasing new electrical appliances. Devices with a better label are often more expensive to purchase, but consume much less energy.

-Always fill the washing drum. This means you have to wash less often and save money! -Wash at 30 degrees as much as possible. The water needs to be heated less, and that saves you a lot of money!

-Halve the number of drying cycles per week. You save €X, XkWh, Xkg

-Do not use a tumble dryer. You save €X, XkWh, Xkg

-Air your home daily when you dry the laundry indoors. Otherwise your home will become damp and mold may develop. That is bad for your health.

**Led lamps**

-Led lamps

-Number ,Wattage,Sort, Saving €

-Replace the old lamps with LED lamps. You save €X, XkWh, Xkg

**Total savings**

-This can save you a total of euros and CO2:

-You can save total energy by doing this:

-You can save total gas by doing this

-You can save total heat by doing this

**9. Free Saving Products**

A. Order List

-Selection, Number of, Saving (€), cost (€)

- -Radiator foil
- -Radiator bleeding key
- -Draft Strip window
- -Draft Strip door
- -Letterbox Insulation
- -Shower Timer
- -Water Tap Limiter
- -Shower head
- -Power Strip
- -Contact Switch
- -LED Lamp (2/4/5 Watts)
- -LED Lamp (9 watts)
- Led Lamp Candle (⅓ watts)
- -Thermometer
- Smart Device

**10. Follow up contact**

TIP! We would like to note some personal information about you. These are used for the following purposes: 1) The savings report will be sent to your email address. If you do not have this or do not want to provide it, it will be sent by post. 2) While completing the questionnaire you may receive services from the Foundation! Woon offered. If you have indicated that you wish to make use of this, and you enter an e-mail address or telephone number, an employee of the !WOON Foundation will contact you. Otherwise, you will have to contact us yourself to use these services. 3) The !WOON Foundation has several newsletters. If you enter an email address, you can sign up for these newsletters when you wish to receive them. 4) The Energy Coach team of the !WOON Foundation may contact you for a short evaluation of the home visit.

1. A. Resident's first name

1. B. Resident's last name

2. A. Do you have a city pass with a green dot?

- Yes
- No

2. B. Have you received an energy allowance?

- Yes
- No

3. Resident's email address

*Resident does not have an email address or does not want to provide one

4. Resident's telephone number

*Resident does not want to provide telephone number

TIP! Do you have any other questions about living at the !WOON Foundation? Then come to one of our consultation hours for free and without obligation. An overview of the consultation locations and times can be found on our website www.wooninfo.nl/contact.

**Smart Energy Display Installation Protocol**

1. Put the dongle into the P1 port (which may be underneath or behind a plastic cap) wait until it goes red and stops flashing. It only works for meters made in 2014 and after. The dongle is paired to that display only so you cannot see your neighbours usage.

2. Put the display within 30m of the smart meter and in a visible place for all household members to see.

3. Choose single or dual rate tariff (depending on if they pay one price through the day or day and night prices. If you cannot find the answer on the bill choose single tarrif. The average tariff for electricity is 40-50 cent per kWh while the average for gas is 120-180 cents per m3. For solar production it is .25kWp per panel.

4.Choose daily standing charge (what the home pays in addition to consumption for being connected to the energy at all times). This can be skipped because the effect of behaviour change can be seen more clearly when only showing current consumption x the rate as costs. The standing charge cannot be influenced. If you do this, show the resident that this display does not apply to fixed costs then.

5. You can turn off all the devices and appliances at the beginning to isolate and see which consumes what. However, there will always be some background consumption such as the fridge. The electricity is on a 1-10 second delay and the gas is 30 minutes in the beginning.

Background consumption will be around 100-300watts. Testing with a kettle or turning off lights is advised.

6. Put the budget lower than what they are currently on, or in any case give them something reasonable to aim for. Usually 5-10% lower is best. The budget setting then accounts for seasonal corrections, where after a week of data collection, it looks back to make a forecast. When calculating the budget high and low usage days are considered, showing when they are at home more.

7. Any of the settings can be changed after and if it gets taken put of power source it will remember all the previous usage.

8. It has a thermometer inside that is sensitive also, so if placed near the window or the oven it will react to that. The desired temperature can be set.

9. Leave the flyers with the in case the homes needs to reach out to tech support.

10. Send them the email on hares, turtles and chameleons. The display is designed to save you at least €100 annually.

**Extra Questions Relating to energy poverty not covered by the organizations protocol**

**Income**

1. What is your income type (or job)?

2. What is the total monthly income of your home?

3. What is the total monthly housing costs of your home?

4. What is the total monthly energy costs of your home?

5. Do you ever experience problems paying your energy bills?

6. What is the total monthly food costs of your home?

**Consumption and costs**

7a. Who is your energy supplier?

7b. Have you even been disconnected from your energy supply?

8a. How up to date and space specific is your consumption feedback?

8b. At what time of the day do you use the most energy?

9. Do you feel you can keep your home adequately cool in Summer and warm in Winter?

10. How have you coped with rising energy prices?

11. Do you knowingly over/under consume energy in your home?

12. Do you ever refrain from activities like cooking to save on money and energy?

**Efficiency**

13. Do you know the energy label of your home/fridge/oven/washing machine?

14. What is the inside temperature of your fridge?

15. What is the size of your home in square metres?

16. Is there any presence of mould, damp or leaks?

**Social Characteristics**

17. What is your age and gender?

18. How long have you lived in the Netherlands for?

19. Do you have any physical or mental health issues in the home?

20. Have energy issues affected your own (or your families) mental well-being at all?

21. Do you feel you have the necessary resources to independently participate in the wider energy transition of Amsterdam?

**Appendix B. Static Email Report for Homes**


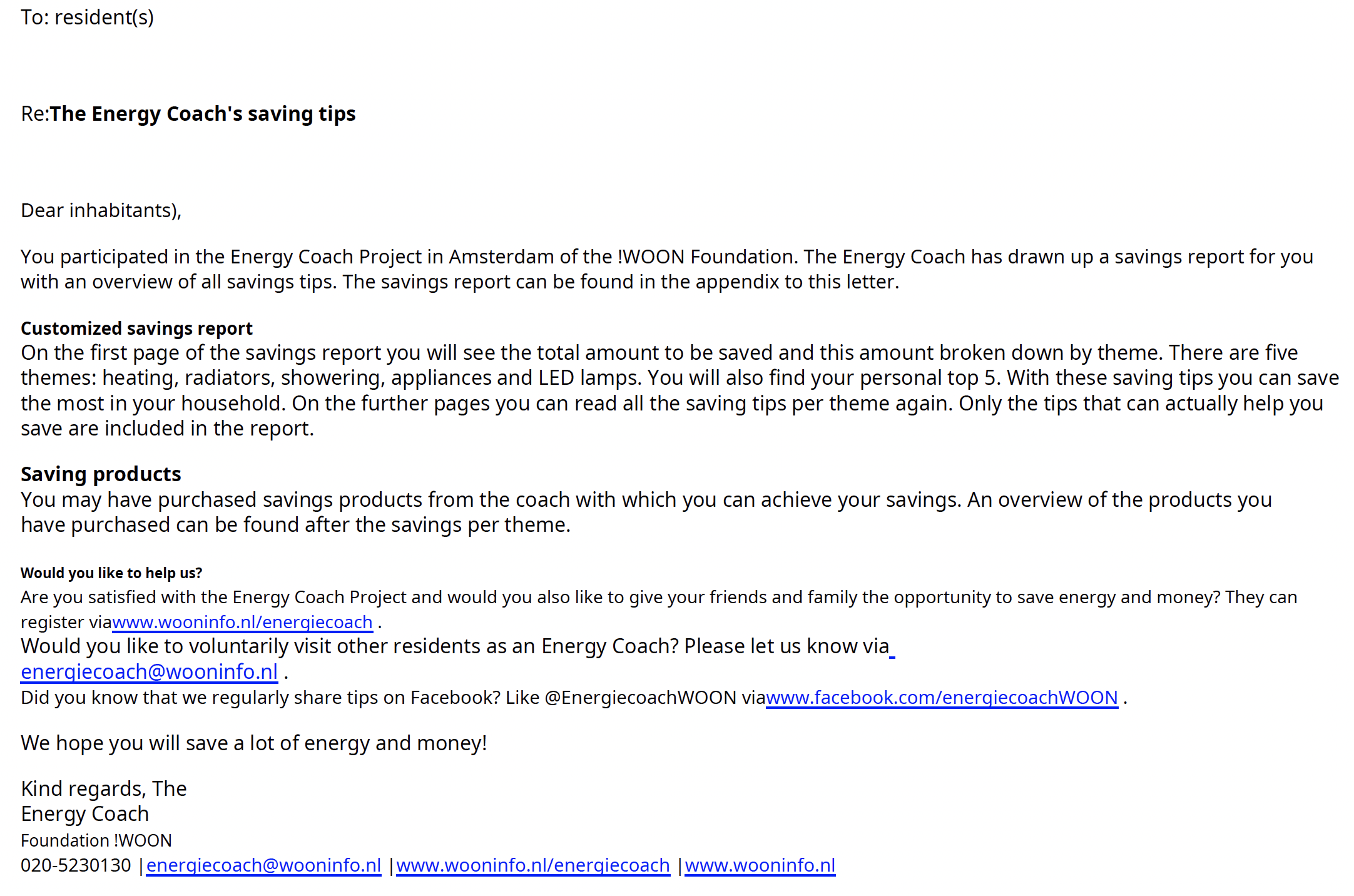


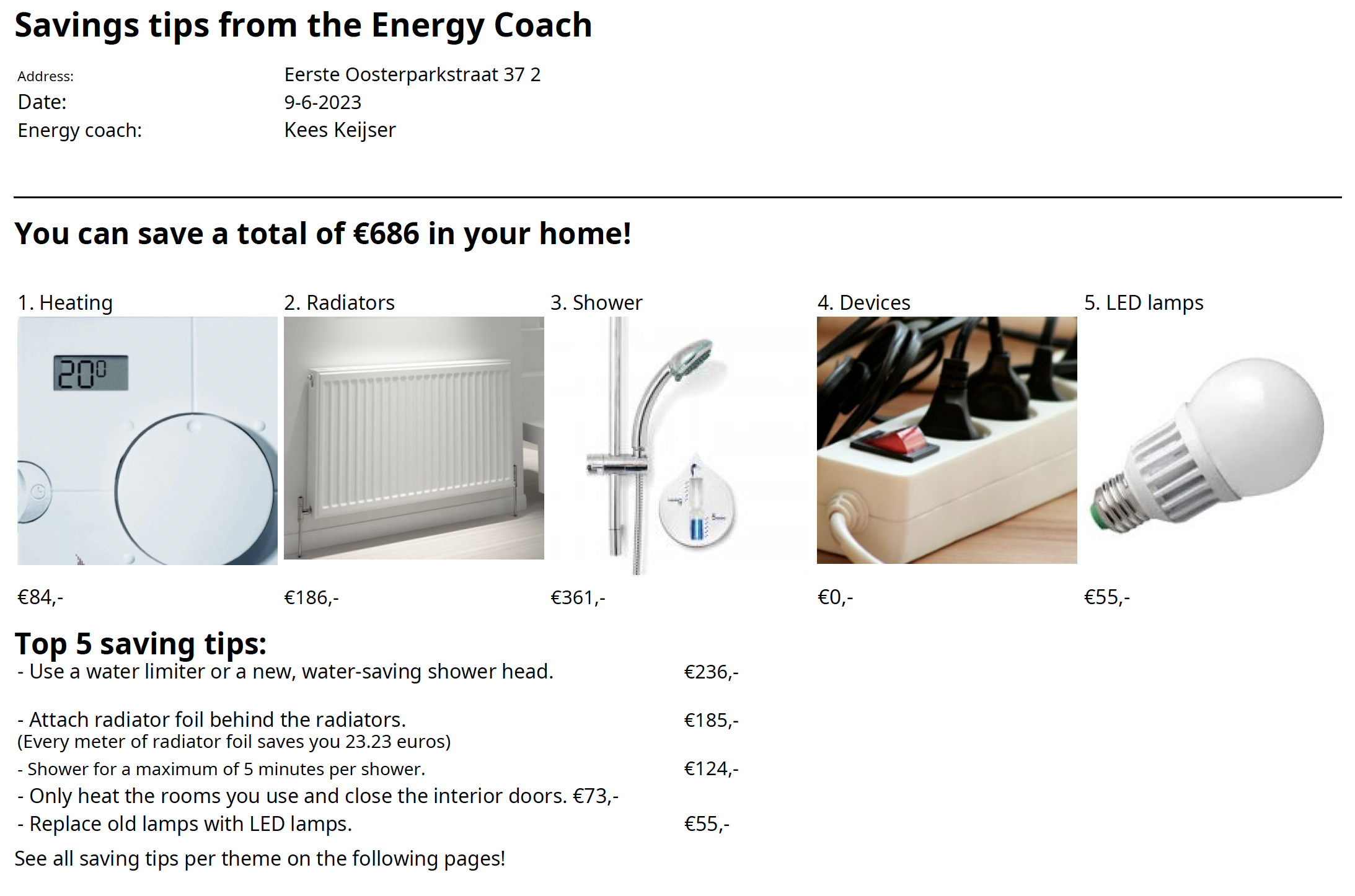


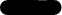


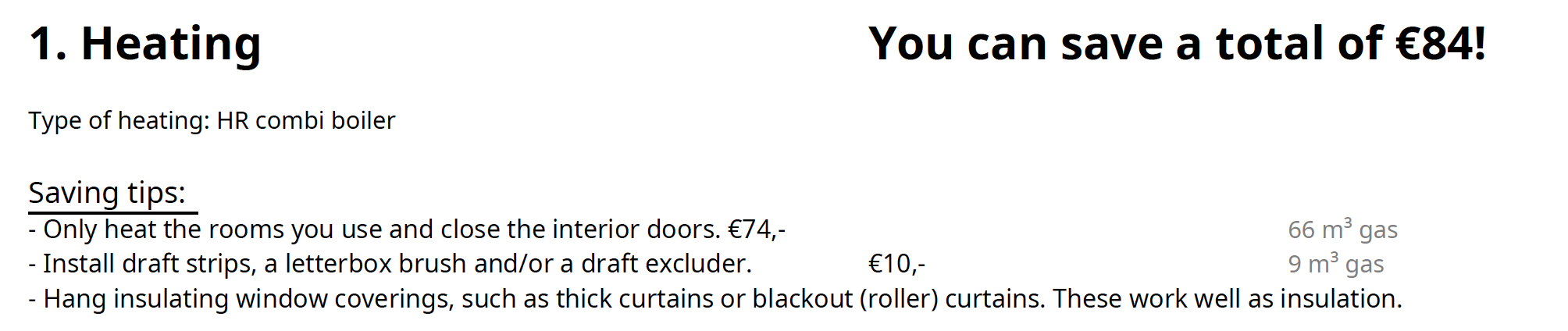


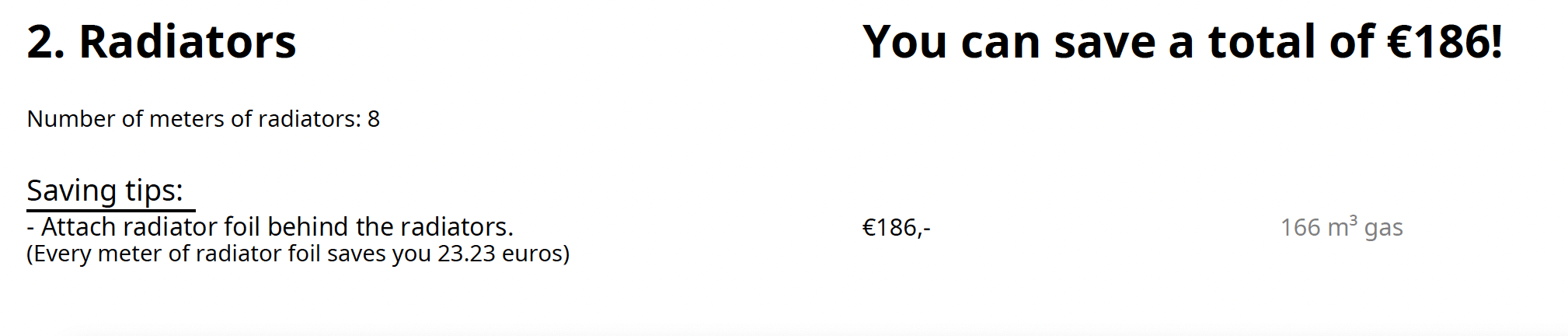


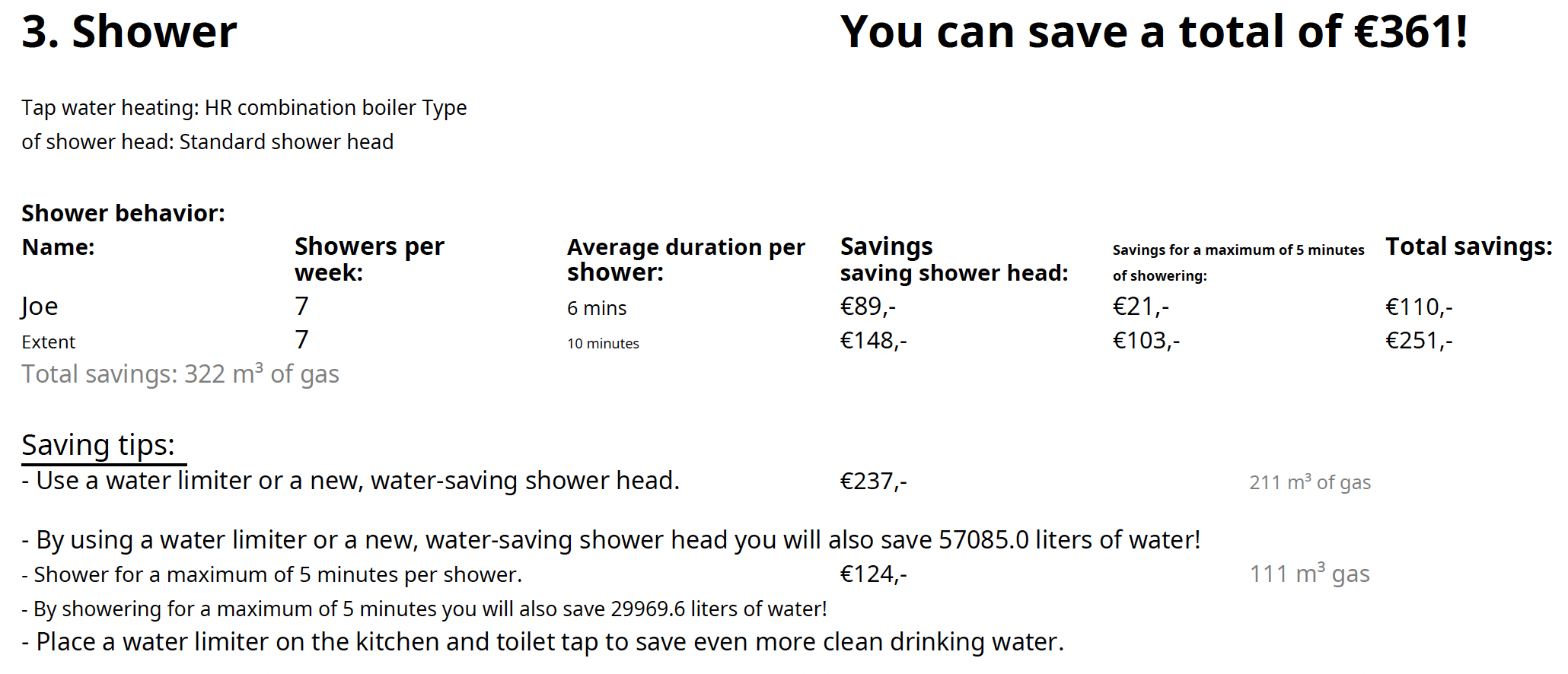


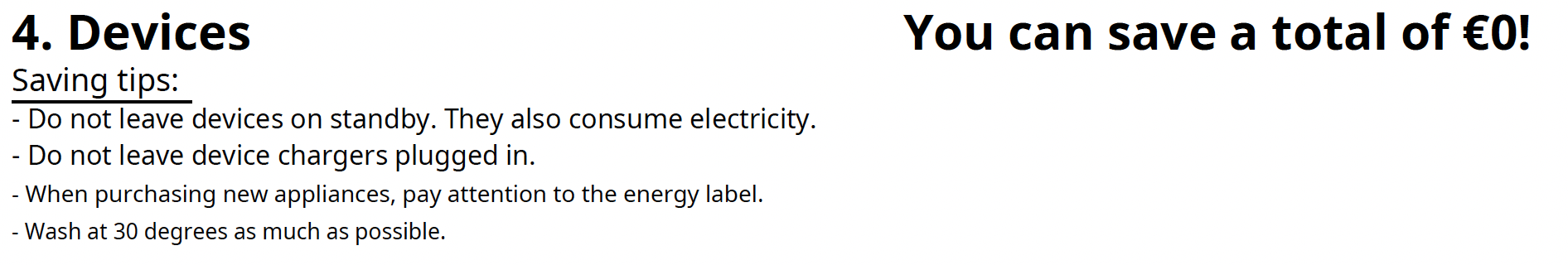


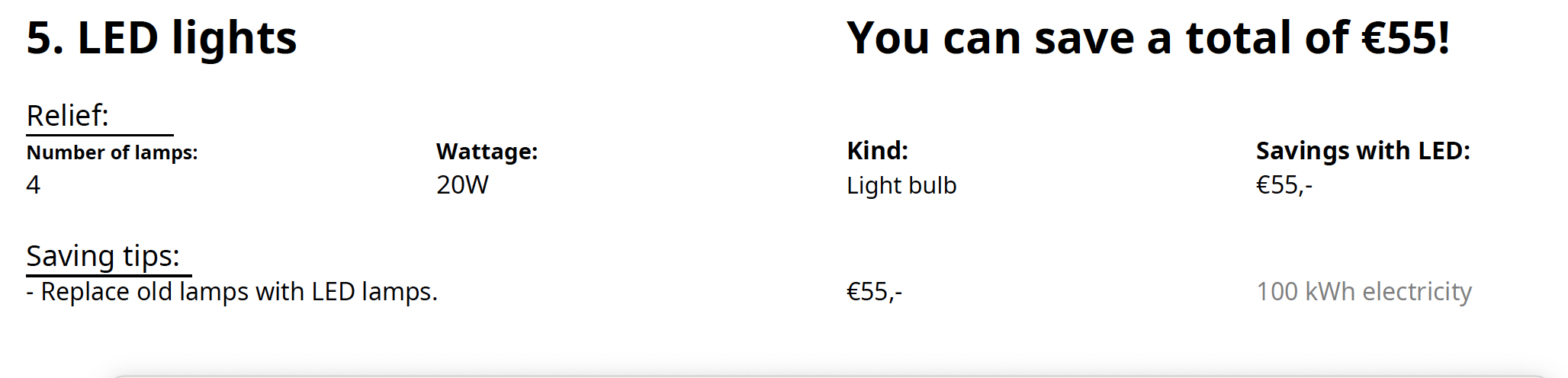


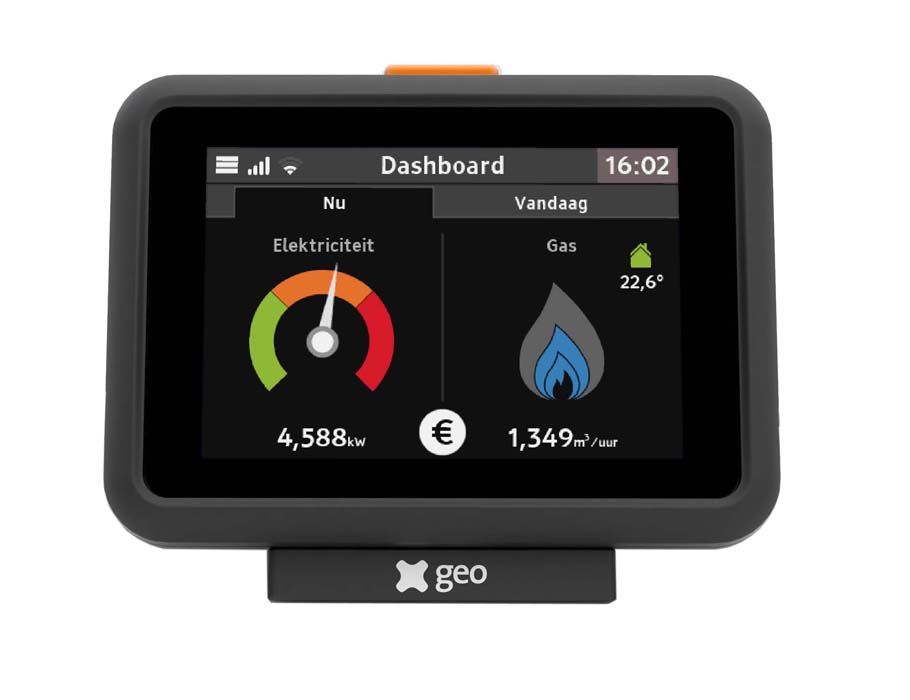
**Appendix C. Home Screen Dashboard for Smart Energy Display**
